# Novel floxed cannabinoid receptor 2 mouse line combines knockout capability with dual fluorescent reporters

**DOI:** 10.3389/fphar.2025.1682979

**Published:** 2025-11-19

**Authors:** Kathryn J. Laloli, Peggy Rentsch, Sandy Stayte, Bryce Vissel

**Affiliations:** 1 Faculty of Medicine and Health, University of New South Wales, Kensington, NSW, Australia; 2 Centre for Neuroscience and Regenerative Medicine, St. Vincent’s Centre for Applied Medical Research, St Vincent’s Hospital Sydney, Sydney, NSW, Australia

**Keywords:** cannabinoid, microglia, knockout, neuroinflammation, mouse

## Abstract

**Background:**

The cannabinoid receptor 2 (CB_2_) is involved in regulating immune responses, yet its specific function in microglia remains poorly defined. This study aimed to generate and validate a microglia-specific, inducible CB_2_ knockout mouse model incorporating reporter genes to enable precise detection of CB_2_ expression and CB_2_ knockout.

**Methods:**

A novel floxed CB_2_ mouse line was generated, incorporating GFP and tdTomato reporter genes driven by the *Cnr2* promoter to indicate CB_2_ expression and CB_2_ knockout, respectively. This line was crossed with Cx3cr1 or Tmem119 tamoxifen-inducible Cre lines to achieve macrophage- or microglia-specific CB_2_ knockout, respectively. Behavioural testing, *in vitro* assays, sequencing and *in vivo* immunofluorescence were used to assess the efficiency and specificity of CB_2_ knockout as well as potential off-target effects.

**Results:**

The floxed allele did not alter breeding or motor behaviour in mice, nor CB_2_ function. CB_2_ expression, indicated by GFP, followed expected patterns across tissues and conditions. Sequencing revealed both DNA and RNA of the floxed allele was as anticipated. Tamoxifen-induced Cre activity successfully initiated tdTomato expression exclusively in microglia of tamoxifen treated, Cre positive mice, validating the specificity and inducibility of CB_2_ knockout. Microglial tdTomato expression confirmed successful CB_2_ knockout in 9.3% of TmemCB_2_ and 91.7% of Cx3CB_2_ microglia. Peripheral tdTomato expression persisted beyond 3 weeks post-tamoxifen in Cx3CB_2_ mice but was minimal in TmemCB_2_ mice.

**Conclusion:**

This novel microglia-specific, inducible CB_2_ knockout model is the first to combine a floxed CB_2_ allele with reporter genes, an essential advancement given the lack of reliable CB_2_ antibodies. The findings demonstrate the model’s specificity and effectiveness, while highlighting important considerations regarding Cre-mediated effects and recombination specificity. Furthermore, the floxed mouse can be crossed with any Cre line to study CB_2_ expression and function in various tissues. This model provides a powerful platform for advancing understanding of CB_2_ roles in microglia and supports future exploration of CB_2_-targeted therapeutic strategies.

## 1 Introduction

The endocannabinoid system (ECS), a tightly regulated signalling network present in all animal species, is a key regulator of homeostasis, including immune responses (Di Marzo, 2018; Van et al., 2021; Silver, 2019; Lowe et al., 2021; Antignano et al., 2023; Rakotoarivelo et al., 2024). The ECS includes cannabinoid receptors CB_1_ and CB_2_, along with their endogenous ligands, endocannabinoids. While CB_1_ is mostly expressed by neurons, CB_2_ is primarily expressed by immune cells. CB_2_ expression is upregulated during inflammation and is understood to play a crucial role in immune modulation (Brusco et al., 2008; Ferranti and Foster, 2022; N. Joshi and Onaivi, 2019). Numerous studies have demonstrated the therapeutic effects of CB_2_ agonists in a large range of disease models, including Alzheimer’s disease (Wu et al., 2013; Jayant et al., 2016; Fakhfouri et al., 2012; C. Li et al., 2019; Aso et al., 2013; Moreno et al., 2012; Sobue et al., 2024), Parkinson’s disease (Rentsch et al., 2020; Gómez-Gálvez et al., 2016; Chung et al., 2016; Liu et al., 2022; Espadas et al., 2020; Viveros-Paredes et al., 2017; H. Yu et al., 2021; Joers et al., 2024), Huntington’s disease (Sagredo et al., 2009; Palpagama et al., 2019), traumatic brain injury (Braun et al., 2018), cerebrovascular and cardiovascular disorders (Yu et al., 2021; Zarruk et al., 2012; Tang et al., 2016; More et al., 2024), metabolic disorders (Rorato et al., 2022; Youssef, El-Fayoumi, and Mahmoud, 2019; Rohbeck, Eckel, and Romacho, 2021; Verty et al., 2015; Rossi et al., 2016; Hosoki, Asahi, and Nozaki, 2024), pain (Xu et al., 2023; Monory and Lutz, 2005; van den Hoogen et al., 2021; Nan et al., 2023), cancer (Alenabi and Malekinejad, 2021; Gambacorta et al., 2023) and more (Smoum et al., 2022; Whiting et al., 2022; Gasperi et al., 2023; Lowe et al., 2021; Grabon et al., 2023a; Onaivi, 2006; Kong et al., 2014; Espejo-Porras et al., 2019). These studies predominantly attribute the therapeutic effects to CB_2_’s anti-inflammatory properties; however, the cell-specific mechanisms mediating these effects remain unclear.

In the central nervous system (CNS), CB_2_ is minimally expressed under basal conditions, but its expression is upregulated in microglia, the resident immune cells of the brain, during neuroinflammation (Duffy et al., 2021). Microglia are essential for maintaining CNS homeostasis and neuronal health. In their resting state microglia continuously monitor the environment for threats. Their phenotypic plasticity allows them to rapidly adapt to environmental cues, and upon activation by inflammatory stimuli, microglia adopt a pro-inflammatory phenotype, a defining feature of neuroinflammation (Araki, Ikegaya, and Koyama, 2021; Prinz, Jung, and Priller, 2019; Umpierre and Wu, 2021; Gao et al., 2023; Paolicelli et al., 2022). Chronic neuroinflammation is implicated in disease pathogenesis, as demonstrated *in vivo* (Wang P et al., 2022; Kang et al., 2024; Penney et al., 2024; Liang et al., 2023; Shi et al., 2019; Dong et al., 2021; Munro et al., 2024; X. Chen et al., 2023; Bido et al., 2021; Gratuze et al., 2023; Arvanitaki et al., 2024; Jing et al., 2021; Ding et al., 2021; Cui et al., 2021; Rocha et al., 2023; W. Kong et al., 2023; Pan et al., 2023; Ryan et al., 2023; Kitchener, Dundee, and Brown, 2023), *in vitro* (Zhou et al., 2023; Salvadores et al., 2022; C. Zhang et al., 2023), and genetic studies (Takatori et al., 2019; Andersen et al., 2021; Corley et al., 2021; ConsortiumInternational Multiple Sclerosis Genetics, 2019; Rodero et al., 2008). Mechanistically, pro-inflammatory microglia contribute to neuronal damage through various pathways, including direct neuronal interactions (Lindhout et al., 2021; Fricker, Oliva-Martin, and Brown, 2012; Butler et al., 2021), the release of neurotoxic cytokines (Lindhout et al., 2021; Rodriguez-Gomez et al., 2020; Oyarce et al., 2022; X. Liu et al., 2021), propagation of pathogenic proteins (Zheng and Zhang, 2021; Wang C et al., 2022; Odfalk, Bieniek, and Hopp, 2022; Xia et al., 2021), and recruitment of peripheral immune cells (Liddelow et al., 2017; X. Chen et al., 2023; Green et al., 2024; Joshi et al., 2019). These pathological activities are compounded by the loss of microglial homeostatic functions (Della Valle et al., 2024; Borst, Dumas, and Prinz, 2021; Zrzavy et al., 2017; Sobue et al., 2021; Kwon and Koh, 2020; Angelova and Brown, 2019). Conversely, microglia in anti-inflammatory states can exert neuroprotective effects, and there is growing evidence that therapies aimed at shifting microglia from a pro-inflammatory to an anti-inflammatory phenotype is beneficial in treating neurodegenerative diseases (Q. Li et al., 2021; Wang W et al., 2023; Willis et al., 2020; Shibuya et al., 2022; Mader et al., 2024; Yoo et al., 2023; Chadarevian et al., 2024; Munro et al., 2024; Daria et al., 2017; Lee et al., 2020; Tao et al., 2021; Jang et al., 2022; Pan et al., 2023; Piano et al., 2023; Birkle and Brown, 2023; Z. Yang et al., 2019; Guo, Wang, and Yin, 2022; Rizzi et al., 2018; Gao et al., 2023). CB_2_ is upregulated in pro-inflammatory microglia, and CB_2_ stimulation has been shown to mitigate their activation, making CB_2_ an attractive therapeutic target (Wang M et al., 2023; Chung et al., 2016; Ojha et al., 2016; Chen et al., 2025).

Although microglia are the principal CB_2_-expressing cells in the CNS, studies have reported CB_2_ expression in certain neuronal populations, other glial cells, and by immune cells that infiltrate the CNS during inflammation (Ziring et al., 2006; Robinson et al., 2015; Jia et al., 2020). However, the extent and functional significance of CB_2_ expression in these cell types remains debated, in part due to challenges in antibody specificity and detection methods (Atwood and Mackie, 2010; Grabon et al., 2023a; Eraso-Pichot et al., 2023). This raises a critical question: Are the therapeutic effects of CB_2_ agonists primarily mediated through microglia, or do other CNS and peripheral cell types contribute significantly?

To address this knowledge gap, we have generated a novel floxed CB_2_ mouse line (CB_2_flx). This model allows for conditional knockout (KO) of the entire coding region of *Cnr2*, the gene encoding CB_2_, in specific cell populations by crossing with appropriate Cre driver lines. This line is novel in that it incorporates dual fluorescent reporter genes for visualising CB_2_-expressing cells and cells in which CB_2_ has been deleted. The incorporation of reporter genes overcomes the limitations posed by a lack of reliable CB_2_ antibodies (Grabon et al., 2023a; Grabon et al., 2023b; Zhang et al., 2019), providing a robust tool for investigating CB_2_ expression and function. This line was then crossed with inducible microglia-specific Cre lines, allowing for precise, cell type-specific deletion of CB_2_ in microglia. Given the complexity of the genetic modifications, rigorous validation is essential to ensure the specificity, efficiency, and functional neutrality of the system prior to its application in disease models.

## 2 Methods

### 2.1 Animals

#### 2.1.1 Cre-Lox FLEx switch mechanism

**FIGURE 1:**
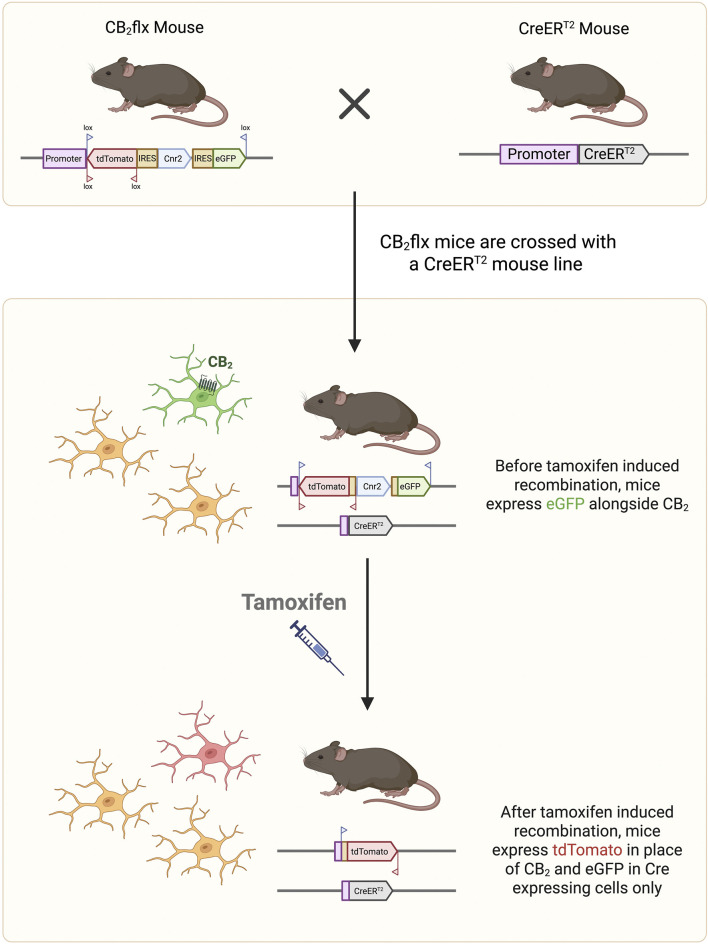
Conditional CB_2_ knockout mechanism. The CB_2_flx mouse line contains genetic modifications in which the entire *Cnr2* coding region (blue box) is surrounded by lox sites. The reporter genes *GFP* (sense orientation) and *tdT* (antisense orientation) are inserted into the *Cnr2* locus under control of the *Cnr2* promoter, such that cells expressing CB_2_ also express GFP. The CB_2_flx line is crossed with a Cre/ERT2 line to create an inducible CB_2_-knockout line. Following tamoxifen injection, Cre mediated recombination excises the floxed *Cnr2* coding region and GFP, while flipping tdT into the sense orientation. Consequently, cells in which CB_2_ has been knocked out now express tdT as a marker of recombination. GFP, green fluorescent protein. tdT, tdTomato.

A spatially and temporally specific CB_2_-KO mouse line was generated using the Cre-Lox recombination system. CB_2_ is encoded by the *Cnr2* gene, which contains a single protein coding exon (exon 2). The entire coding region of *Cnr2* was flanked by loxP sites using homologous recombination in embryonic stem cells, enabling complete KO of CB_2_ protein expression upon Cre-mediated recombination. To facilitate monitoring of *Cnr2* expression and recombination, *GFP* and *tdTomato* (*tdT*) reporter genes were inserted under the control of the *Cnr2* promoter. The *tdT* cassette was placed in an inverse orientation and flanked by lox2272 sites as part of a flip-excision switch (Supplementary Figure S1A). In the absence of Cre recombinase, CB_2_ and GFP are expressed under the control of the *Cnr2* promoter. Following Cre-mediated recombination, the floxed *Cnr2* coding region and *GFP* are excised, while *tdT* is simultaneously flipped into the sense orientation and will now be expressed under the *Cnr2* promoter, providing a permanent marker of recombination (Figure 1; Supplementary Figure S1B). This novel line was named C57BL/6-*Cnr2* ^tm1(GFP,tdTomato)BViss^/J line (CB_2_flx).

Genotyping of all genetically modified mice was performed for the *Cnr2* floxed allele and relevant *Cre* alleles using real-time PCR with SYTO9-based melt curve analysis on a LightCycler 480 system. Primer sequences and PCR conditions are detailed in Supplementary Table S1.

#### 2.1.2 Housing and husbandry

Mice were housed under specific-pathogen-free conditions at 21 °C ± 1 °C with a 12-h light-dark cycle, in plastic cages with *ad libitum* access to water and standard chow. A maximum of five same-sex mice were housed per cage. Mice were acclimated to the facility for at least 1 week prior to experiments. Experimental groups comprised 8–12-week-old male and female mice, and mice of the same sex and genotype were randomly assigned to experimental groups.

All genetically modified mice used in experiments were homozygous for the floxed *Cnr2* allele. Experimental TmemCB_2_ and Cx3CB_2_ mice were bred so that littermates were either heterozygous (Cre/+) or wildtype (+/+) for the Cre/ERT2 allele.

All animal research and care procedures were approved by the Garvan Institute/St. Vincent’s Animal Ethics Committee (ARA numbers 23/13, 20/10, 18/37), per guidelines issued by the National Health and Medical Research Council of Australia and the Australian Code of Practice for the Care and Use of Animals for Scientific Purposes.

### 2.2 Tamoxifen administration

Tamoxifen (Tam) (20 mg/mL) was dissolved in sunflower oil. Mice received i.p. injections of Tam or vehicle control according to three regimens: 150 mg/kg every other day for four doses, 100 mg/kg daily for 8 days, or 100 mg/kg twice daily for ten doses. Tissue was collected 3 weeks after the final injection.

### 2.3 Motor behaviour

Locomotor activity was assessed in a 30 × 30 cm plexiglass open field arena within a sound-attenuated chamber under dim illumination (MedAssociates). One day following a 10 min habituation session, mouse movements were tracked for 10 min. Total distance travelled, centre zone (19 × 19 cm) entries, and time spent in the centre zone were recorded. Blinding was maintained during testing.

Mice were trained on an accelerating rotarod (0–40 RPM) whereby they were placed on the rotarod and returned to it each time they fell off, for 5 min. The following day, latency to fall was measured over three 5-min trials, separated by 30-min intervals. The average of the highest two trials was used for analysis. Experimenters were blinded to treatment groups.

### 2.4 Stereotaxic lipopolysaccharide injection

To induce inflammation-induced neurodegeneration, stereotaxic surgery was performed to ipsilaterally inject the striatum with the endotoxin lipopolysaccharide (LPS). Intrastriatal LPS is well established to produce a robust neuroinflammatory response in the substantia nigra (SN), while its large volume allows for more accurate targeting and reduced mechanical damage (Deng et al., 2020; Jang et al., 2022; Skrzypczak-Wiercioch and Salat, 2022). Before surgery, mice received 4 × 150 mg/kg doses of Tam or vehicle control, once every other day, followed by a 4-week washout period. Mice were then anesthetised via an anaesthetic cocktail (10 mL/kg, i.p.), containing ketamine (Mavlab, Australia; 10 mg/mL) and xylazine (Troy Laboratories, Australia; 2 mg/mL). LPS was administered as previously described (Gómez-Gálvez et al., 2016). Briefly, LPS (*Salmonella enterica*, Minnesota) was dissolved in 0.9% sterile saline to a concentration of 5 mg/mL. Two injections of 1 μL of LPS were injected into the right striatum at AP +1.18, ML -1.5, DV -3.5 and AP -0.34, ML -2.5, DV -3.2, relative to bregma and the dural surface. Injections were given at a rate of 0.5 μL/min using a 2 μL Neuros syringe (Hamilton, Germany).

Mice were acrificed for histological analysis 15 days after surgery.

### 2.5 Histology

#### 2.5.1 Tissue collection and processing

Animals were anaesthetised before cardiac perfusion with 4% paraformaldehyde. The brain was immediately dissected out and submerged in paraformaldehyde at 4 °C for 24 h, before being transferred to cryoprotectant solution (30% sucrose in PBS).

#### 2.5.2 Immunofluorescence

Free-floating 40 µm sections were blocked in 3% BSA +0.25% Triton for 1 h before primary antibody (Supplementary Table S2) was applied for 72 h, at 4 °C. Tissue was then incubated with the corresponding Alexa Fluor-conjugated secondary antibody for 24 h at 4 °C, protected from light. Finally, the sections were incubated in DAPI (Thermo Fisher; 1:1000 in PBS) for 10 min.

#### 2.5.3 FIJI image analysis

For colocalization of GFP, tdT and Iba1, four representative images of each brain were taken in consistent areas of the striatum, SN, and hippocampus with a Zeiss Axio Imager.Z2 microscope. For each image, four z-stacks were taken using the ×40 objective. Percentages of GFP, tdT and Iba1 colocalization were quantified using the FIJI (ImageJ) Colocalization Object Counter plugin (Lunde and Glover, 2020). Both the max projection and stack were analysed. A cell would only be counted if the nuclear marker DAPI was present.

### 2.6 *In vitro* cAMP assay

Primary glia cultures were prepared from P0–P3 C57BL/6JAusb (BL6), CB_2_flx and Cx3CB_2_ pups, as previously described (Schildge et al., 2013). Briefly, cortices were dissected in dissection medium (HBSS, 1% P/S) dissociated with trypsin-EDTA (0.005%), and a single cell suspension in glia medium (DMEM/F12, 10% FBS, 1% P/S) was added to PDL coated flasks. Media was changed after 24–36 h and then every 2–3 days thereafter, until the cells reached approximately 90% confluence (∼9 days). Microglia and astrocytes were separated by shaking cultures at 200 RPM for 6 h. Microglia and astrocyte cell pellets were resuspended in stimulation buffer (HBSS + 5 mM HEPES +0.5 mM IBMX +0.075% BSA; pH 7.4) for use in cAMP assays. Forskolin-stimulated cAMP levels were measured using the LANCE Ultra cAMP kit (PerkinElmer), following manufacturer’s instructions.

For assay optimisation, forskolin concentration-response curves were established for both microglia and astrocytes at various cell densities to determine the optimum cell density and forskolin concentrations for subsequent CB_2_ agonist cAMP assays. It was concluded that the ideal conditions for microglia were 2000 cells/well with a forskolin concentration of 71.4 µM (Supplementary Figure S2A). For astrocytes, 500 cells/well with a forskolin concentration of 2.6 µM (Supplementary Figure S2B).

For the agonist assays, Hu308 was diluted in DMSO to generate a concentration response curve of forskolin-stimulated cAMP levels for each cell type. For all assays, samples were run in triplicate and a cAMP standard curve was run on every plate. TR-FRET signal was measured with the PHERAstar FSX microplate reader (BMG LabTech).

### 2.7 Statistics

All statistics were performed using Prism 10 software (GraphPad). Unless otherwise stated, *p* < 0.05 was considered significant and data are reported as mean ± standard error of the mean. Before undergoing parametric tests, D'Agostino-Pearson normality test was carried out and if a dataset failed this test, the Q-Q plot was inspected to determine if there were major violations of a Gaussian distribution. Further, Spearman’s rank test was used to test for homoscedasticity, and if this test failed, the residual plot was inspected for major violations. Data that passed these tests underwent one-, two- or three-way ANOVAs. For *post hoc* corrections, Tukey’s was used when comparing every mean with every other mean, Dunnett’s for comparing every mean to a control mean, Dunn’s correction for non-parametric comparisons, and Šídák’s correction for all other *post hoc* comparisons.

Details of all mouse lines, reagents, equipment and software used are listed in Supplementary Table S3.

## 3 Results

### 3.1 Validation of *Cnr2* gene targeting

To validate the genetic modifications in the floxed *Cnr2* locus, DNA sequencing was performed by OzGene. As expected, the sequencing results confirmed alignment with the anticipated sequence across the targeted region, except for a 510 bp stretch within the 5′homology arm, for which incomplete sequencing data were obtained due to poor trace quality. Despite this limitation, the low-quality sequenced portion of the region exhibited 100% identity to the predicted sequence (Supplementary Figure S3). Furthermore, sequencing of *Cnr2* mRNA, performed by The Garvan Institute’s Genetics Core Facility, indicating precise alignment with the anticipated sequence and therefore no unintended disruptions in *Cnr2* transcription. These in-depth sequencing analyses confirm the successful incorporation of the intended genetic modifications in the floxed *Cnr2* locus, with no evidence of off-target alterations affecting *Cnr2* transcription. Full sequencing data for both DNA and mRNA is available on GenBank.

### 3.2 Genetic modifications do not impair reproductive fitness

**FIGURE 2:**
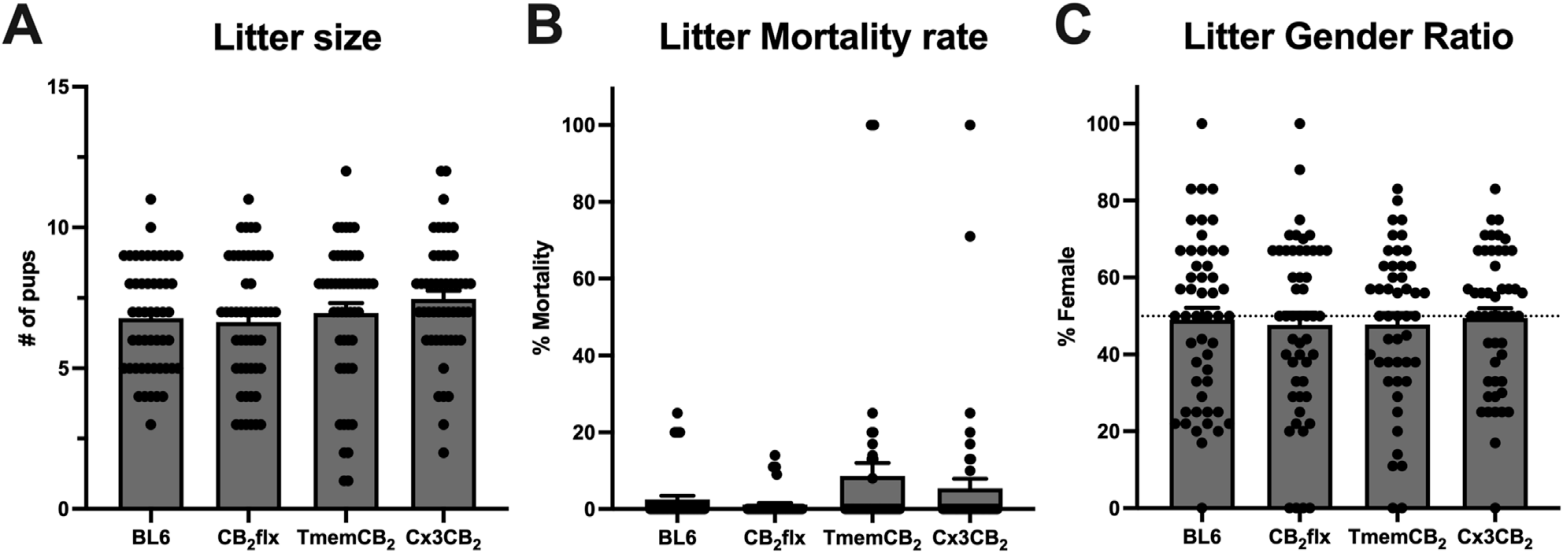
Litter characteristics are not impaired in genetically modified strains. **(A)** Number of pups born per litter. One-way ANOVA, Dunnett’s *post hoc* test. **(B)** Proportion of litter mortality between birth and weaning. One-way ANOVA, Dunnett’s *post hoc* test. **(C)** Proportion of pups that were female at weaning. One-way ANOVA, Dunn’s *post hoc* test. Data were collected from 50 litters per strain over a similar timeframe. Data are represented as mean ± SEM.

The reproductive fitness of the genetically modified mouse lines was assessed compared to BL6 controls by a prospective analysis of breeding outcomes. There was no significant difference between groups for litter size (*F* (3, 196) = 1.31, *p* = 0.27; Figure 2A), mortality rate (*KW*(4, 200) = 3.79, *p* = 0.28; Figure 2B), or sex ratio (*F* (3, 192) = 0.094, *p* = 0.96; Figure 2C). Furthermore, no overt developmental or behavioural abnormalities were observed during routine breeding and handling. These findings demonstrate that the genetic modifications in the CB_2_flx, Cx3CB_2_, and TmemCB_2_ mouse lines do not adversely affect reproductive capacity or offspring viability.

### 3.3 Motor behaviour is unaffected in CB_2_flx mice

**FIGURE 3:**
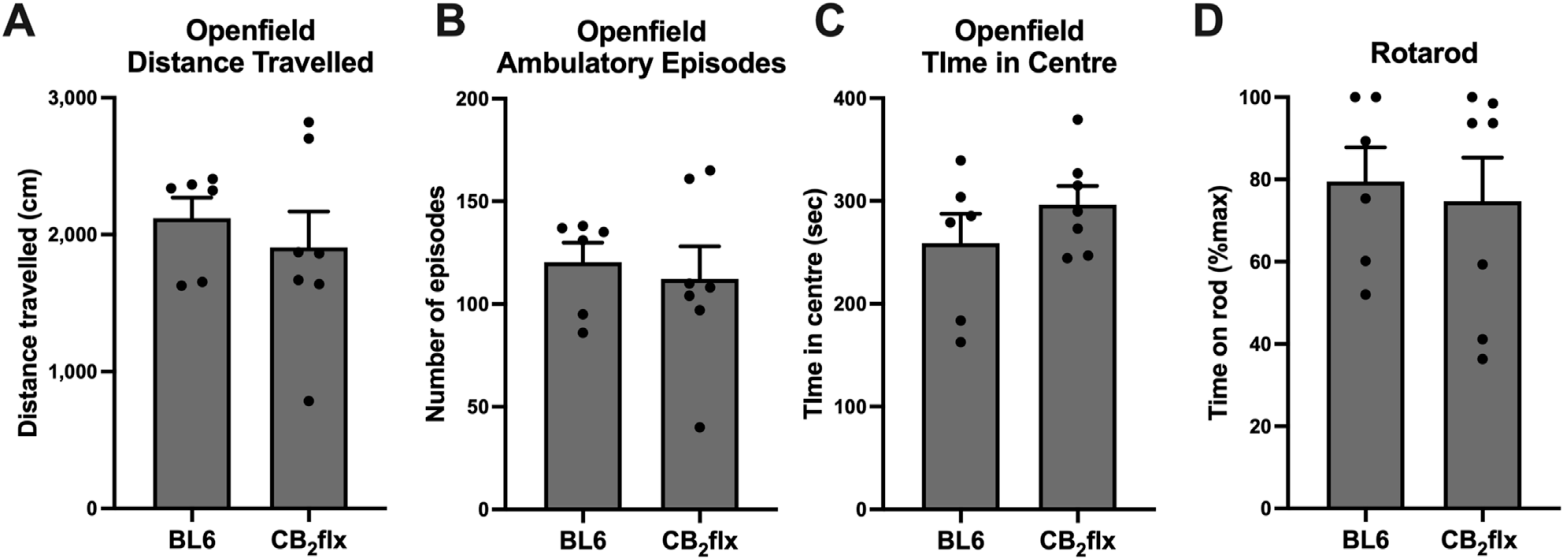
Motor behaviour is not impaired in the CB_2_flx strain. **(A–C)** Open field and **(D)** rotarod motor behaviour tests in CB_2_flx mice. N = 6-7/group. Unpaired t-test. Data are represented as mean ± SEM.

To assess the effects of the floxed *Cnr2* allele on motor behaviour, CB_2_flx mice were subjected to open field and rotarod test. In the open field test, no significant differences were observed between CB_2_flx and BL6 mice in total distance travelled (*t* (11) = 0.670, *p* = 0.516; Figure 3A), number of ambulatory episodes (*t* (11) = 0.421, *p* = 0.682; Figure 3B), or time spent in the central zone (*t* (11) = 1.138, *p* = 0.279; Figure 3C). Similarly, performance on the rotarod test revealed no differences in latency to fall (*t* (11) = 0.346, *p* = 0.736; Figure 3D). Collectively, these findings demonstrate that the presence of the floxed *Cnr2* allele does not affect locomotor activity or motor coordination.

### 3.4 CB_2_ function is preserved in glial cells

As a G_⍺i_-coupled receptor, CB_2_ activation inhibits cAMP production (Kaminski, 1998). Therefore, a cAMP assay was performed to assess receptor function in primary glial cultures from CB_2_flx and Cx3CB_2_ mice. First, optimisation experiments were conducted to determine appropriate cell densities and forskolin concentrations for both microglia and astrocytes (Supplementary Figure S2).

**FIGURE 4:**
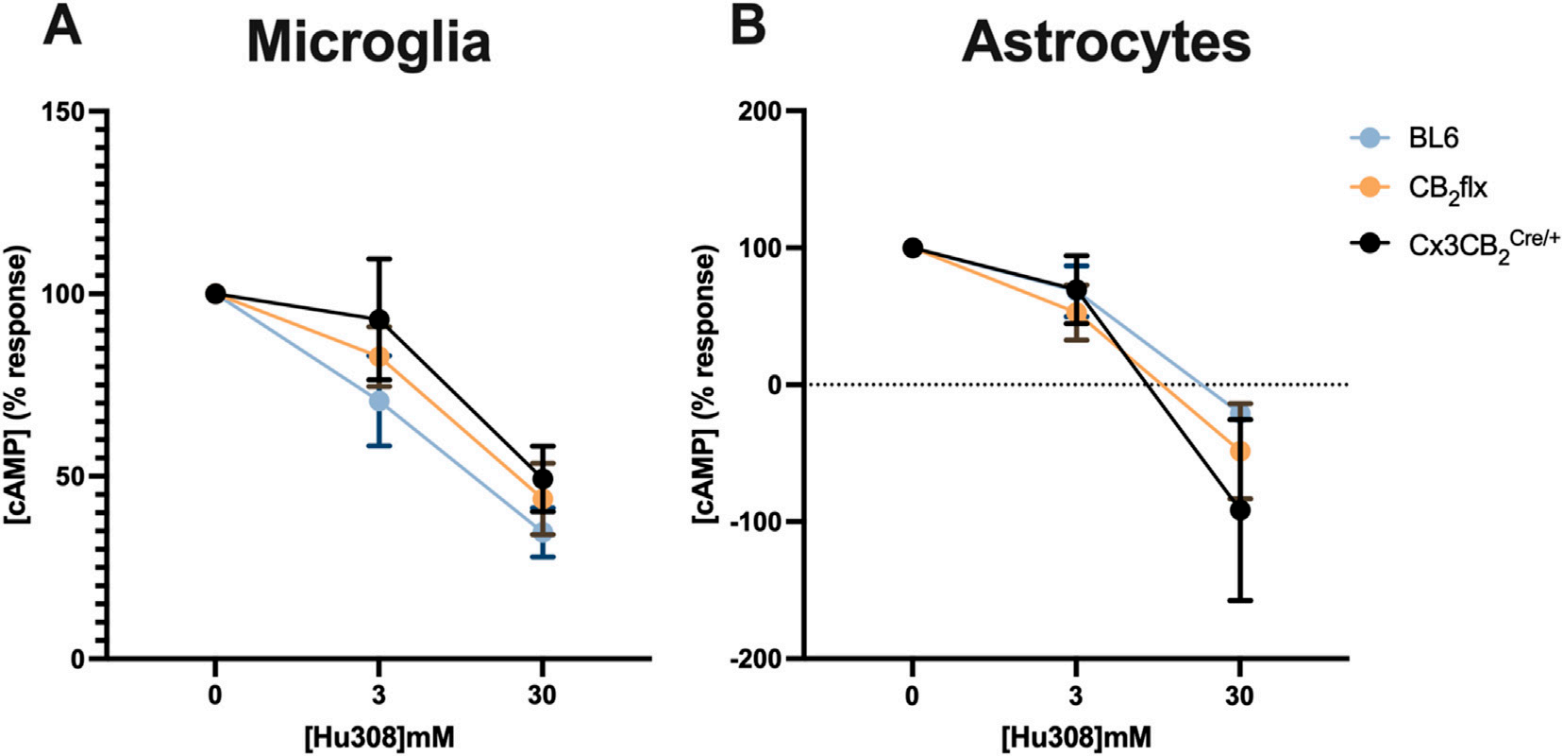
CB_2_ function is conserved in primary glia cultures. The CB_2_ agonist Hu308 inhibited cAMP production in primary **(A)** microglia and **(B)** astrocytes in a dose dependent manner in all strains. Data was normalised whereby 100% represents 0 mM Hu308 and 0% represents no forskolin control. N = 5-8/group. Two-way ANOVA, Dunnett’s *post hoc* test. Data are represented as mean ± SEM. Each point represents the mean of three technical replicates.

Upon stimulation with the CB_2_ agonist Hu308, forskolin-induced cAMP levels were reduced in a dose-dependent manner across all groups. No significant differences were observed in Hu308-induced cAMP inhibition between CB_2_flx, Cx3CB_2_, and BL6 controls for microglia (*F* (2,28) = 0.027, *p* = 0.973; Figure 4A) or astrocytes (*F* (2,32) = 0.432, *p* = 0.653; Figure 4B). These results indicate that CB_2_ function is preserved in glial cells derived from genetically modified strains.

### 3.5 Reporter gene expression in the CB_2_flx mouse line

Given the challenges in using immunohistochemistry to detect CB_2_, a fluorescent reporter system was incorporated into the CB_2_flx line to enable visualisation of CB_2_ expression. In the CB_2_flx line, GFP expression is driven by the *Cnr2* promoter, allowing detection of CB_2_-expressing cells through histological analysis. In the absence of Cre-mediated recombination, tdT should not be expressed. To confirm this, GFP and tdT expression were examined using immunofluorescence in the periphery, as well as in the brain under naive and inflammatory conditions.

**FIGURE 5:**
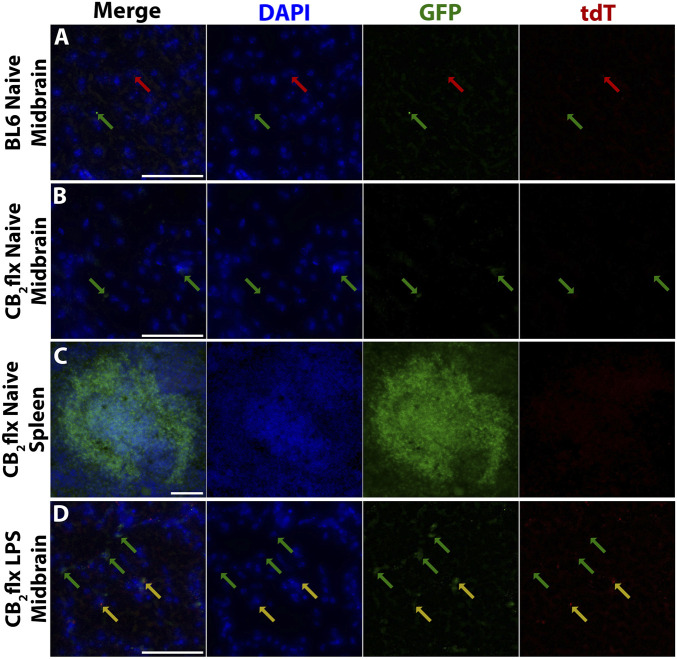
Reporter gene expression reflects expected CB_2_ expression in CB_2_flx mice. Representative images of green fluorescent protein (GFP) and tdTomato (tdT) reporter gene expression in **(A)** the naive BL6 CNS, **(B)** naive CB_2_flx CNS, **(C)** naive CB_2_flx spleen, and **(D)** CB_2_flx CNS treated with intrastriatal LPS. Green arrows = GFP expression. Red arrows = tdT expression. Yellow arrows = GFP/tdT colocalization. Scale bar = 100 µm.

BL6 controls displayed some small points of autofluorescence for both GFP and tdT, which served as an essential benchmark to distinguish true signal from artefacts (Figure 5A). In the naive CB_2_flx brain (Figure 5B), minimal GFP expression was observed, consistent with the low basal expression of CB_2_ in the CNS, while the spleen exhibited strong GFP expression, particularly in white pulp regions, which are enriched in CB_2_ expressing immune cells (Figure 5C) (Galiègue et al., 1995; Simard et al., 2022; Lewis, Williams, and Eisenbarth, 2019).

To assess inflammation-induced CB_2_ expression, neuroinflammation was elicited via intrastriatal injection of LPS. GFP expression increased markedly in the inflamed CNS, aligning with the expected upregulation of CB_2_ during neuroinflammation. Occasional tdT expression was observed, but this was restricted to regions with intense GFP signals, suggesting fluorescence channel bleed-through rather than true tdT expression (Figure 5D).

Overall, GFP expression in CB_2_flx mice mirrored the expected CB_2_ expression profile: low in the naive CNS, elevated in the CNS during inflammation, and pronounced in peripheral tissues. These findings validate the utility of this mouse model for studying CB_2_ expression and function.

### 3.6 Tam induces microglial specific tdT expression in Cx3CB_2_ mice

Given that CNS CB_2_ is primarily expressed by microglia (Duffy et al., 2021), the CB_2_flx line was crossed with a macrophage-specific Cre line (JAX, strain#: 021160), resulting in the establishment of the Cx3CB_2_ line. In these mice, Cre expression is limited to Cx3cr1-expressing cells, which include microglia and peripheral macrophages (Subbarayan et al., 2022; Mizutani et al., 2012). Upon Tam administration, Cre-mediated CB_2_-KO is expected in all Cx3cr1-expressing cells.

To determine the most efficient Tam protocol for inducing Cre-mediated recombination in Cx3CB_2_ mice and to assess the effects of Cre and Tam on CB_2_ expression, we analysed reporter gene expression in BL6, CB_2_flx, and Cx3CB_2_ mice. In Cx3CB_2_ mice heterozygous (Cre/+) for the Cre allele, GFP serves as a marker of CB_2_ expression, while yellow fluorescent protein (YFP) is expressed in Cx3cr1-Cre cells as part of the Cre driver line. A technical limitation of this experimental design is that the overlapping emission spectra of GFP and YFP could not be distinguished using our immunofluorescence approach. Consequently, in Cx3CB_2_^Cre/+^ microglia, which express both YFP (from the Cre allele) and potentially GFP (from CB_2_ expression), the fluorescent signal primarily reflects YFP from Cre expression, and prevents direct assessment of CB_2_-driven GFP in these cells. In contrast, cells from other genotypes (CB_2_flx, Cx3CB_2_^+/+^) and non-microglial cell types do not express YFP, allowing GFP signal in these groups to be unambiguously attributed to CB_2_ expression. Despite this limitation in directly visualising CB_2_ expression in Cre-positive microglia, the tdT reporter system provides definitive confirmation of successful Cre-mediated CB_2_ deletion in these cells.

**FIGURE 6:**
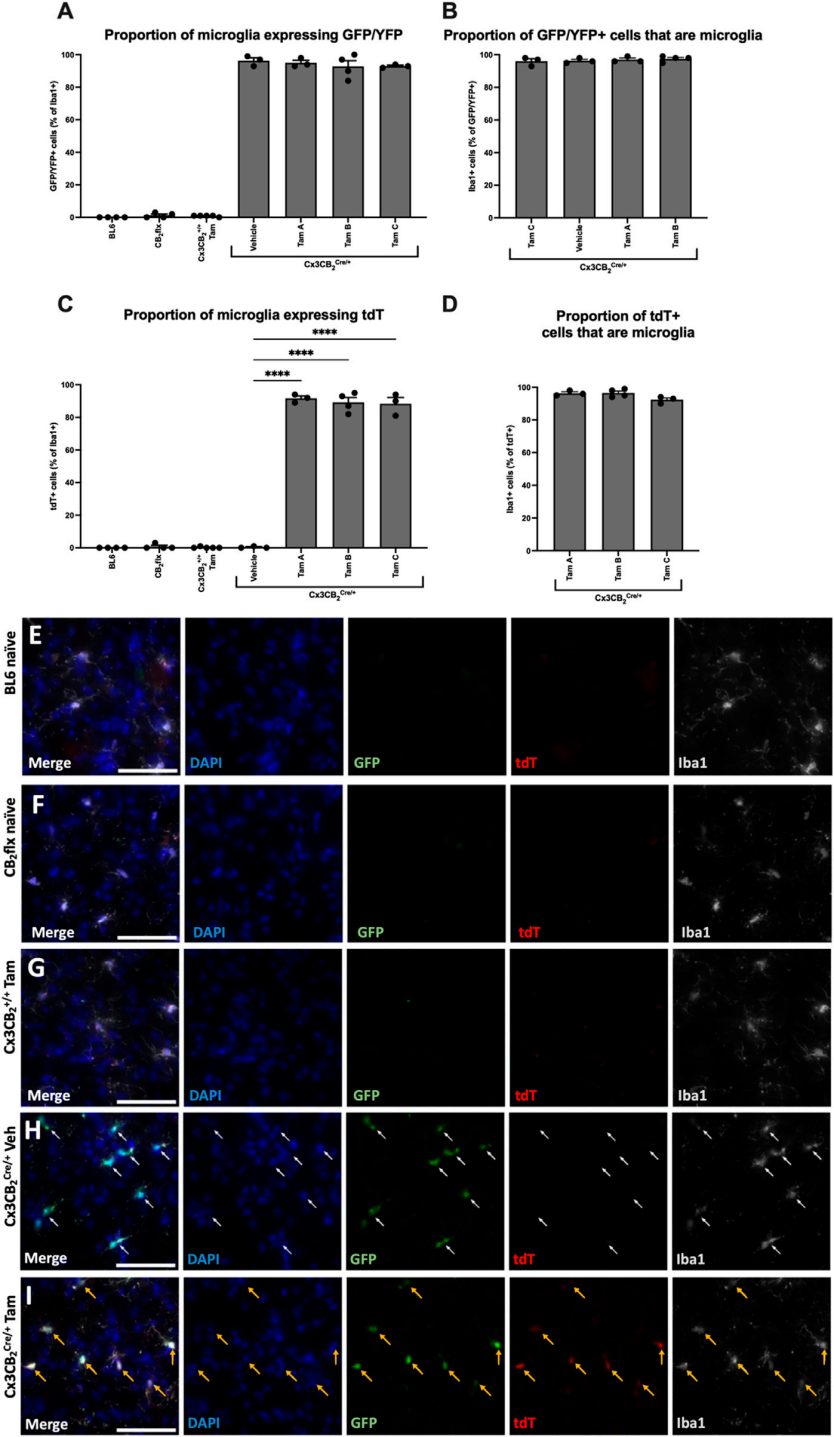
In Cx3CB_2_ mice, tdT expression is microglia specific and is Tam/Cre dependent. **(A–D)** Quantification of GFP and tdT colocalization with Iba1. Significance bars between Cx3CB_2_^Cre/+^ groups only are shown. N = 3-4/group. An average of 133.4 (±4.3) Iba1+ cells were counted per n. Data are represented as mean ± SEM. One-way ANOVA with Tukey’s *post hoc* test, ****p ≤ 0.0001. Representative images of substantia nigra immunostaining in **(E)** BL6, **(F)** CB_2_flx, **(G)** Tam treated Cx3CB_2_^+/+^, **(H)** Vehicle-treated Cx3CB_2_^Cre/+^ and **(I)** Tam-treated Cx3CB_2_^Cre/+^ animals. White arrows = GFP expression. Yellow arrows = GFP/tdT colocalization. Scale bar = 50 µm.

In Cx3CB_2_^Cre/+^ mice, nearly all Iba1+ microglia (>92%) expressed GFP/YFP, with no significant differences between treatment groups (*F* (6, 19) = 923.3, *p* < 0.0001; Figure 6A). Furthermore, over 96% of GFP/YFP signal colocalized with Iba1+ microglia, again with no significant differences between treatment groups (*F* (3, 9) = 0.413, *p* = 0.747; Figure 6B). These results confirm that Cre expression is restricted to microglia in Cx3CB_2_^Cre/+^ mice, and that Tam treatment does not influence Cre expression levels.

Microglia from all other genotypes exhibited minimal GFP/YFP signal, confirming the absence of Cre and supporting prior findings of low basal CB_2_ expression in microglia. Additionally, no significant differences were observed between Tam-treated Cx3CB_2_^+/+^ and BL6 controls, demonstrating that Tam administration does not induce CB_2_ expression in microglia (Figure 6A).

tdT expression serves as a reporter for successful CB_2_-KO in Cx3CB_2_ mice, therefore tdT should be restricted to microglia in Tam-treated and Cre/+ mice. As expected, no significant tdT expression was detected in any control group, confirming the absence of CB_2_-KO. In contrast, nearly all Iba1+ microglia in Tam-treated Cx3CB_2_^Cre/+^ mice expressed tdT (Figure 6C). Within these groups, over 92% of tdT expression colocalized with microglia, with no significant differences between Tam administration protocols, confirming high recombination efficiency across all protocols (*F* (2, 7) = 4.07, *p* = 0.067; Figure 6D). High-magnification (Figures 6E–I) and low-magnification (Supplementary Figure S4) representative images further emphasise the stark differences in reporter gene expression between groups. Furthermore, no GFP/YFP or tdT signal was observed in neurons or astrocytes, confirming that Cre expression, CB_2_ expression, and CB_2_-KO were microglia/macrophage-specific (Supplementary Figure S5).

Given that Cx3cr1-expressing macrophages are also present in peripheral tissues, we next examined CB_2_ expression outside the CNS. Peripheral macrophages have been reported to undergo renewal every 3 weeks, suggesting that tdT should be absent in the spleen at this timepoint, rendering CB_2_-KO microglia-specific thereafter (Wang et al., 2020; Dick et al., 2019; Goldmann et al., 2013; Peng et al., 2016; Bedolla et al., 2023). As expected, Tam-treated Cx3CB_2_^+/+^ spleens exhibited GFP but lacked tdT, similar to CB_2_flx controls (Supplementary Figure S6A). Conversely, tdT was detected in Cx3CB_2_^Cre/+^ spleens 1-week post-Tam, as anticipated (Supplementary Figure S6B). However, tdT expression persisted at 3 weeks, contrary to prior reports, suggesting that CB_2_-KO is not specific to microglia at this timepoint (Supplementary Figure S6C). This finding challenges the conventional assumption that a 3-week waiting period is sufficient to ensure microglia specificity in this Cre line.

These results confirm the microglia-specific expression of Cre in the CNS of Cx3CB_2_^Cre/+^ mice and its Tam-dependent activity. All Tam protocols efficiently induced CB_2_-KO in macrophages, with no significant differences between protocols. Based on these results, four doses of 150 mg/kg administered every other day was selected for continued use, as it exhibited the highest mean microglial tdT expression and specificity of 91.7%. Notably, CB_2_-KO persisted in peripheral macrophages beyond 3 weeks post-Tam, highlighting the need for extended evaluation periods if exclusive microglial specificity is required in this model.

### 3.7 Tamoxifen induced reporter gene expression in the TmemCB_2_ line

To improve microglia specificity of the CB_2_-KO, we generated the TmemCB_2_ line by crossing the CB_2_flx line with a Tmem119^Cre/ERT2^ line (JAX, strain# 031820), in which Cre expression is driven by the highly microglia-specific Tmem119 promoter (Kaiser and Feng, 2019). We assessed CB_2_-KO efficiency and specificity in the TmemCB_2_ line using the optimised Tam protocol established in Cx3CB_2_ mice (four doses of 150 mg/kg administered every other day). Tam was administered to TmemCB_2_ mice either homozygous for the wild-type *Tmem119* allele (+/+) or heterozygous for the Tmem119^Cre/ERT2^ allele (Cre/+), alongside untreated BL6 controls. Only microglia in the Tam-treated TmemCB_2_^Cre/+^ group were anticipated to express tdT.

**FIGURE 7:**
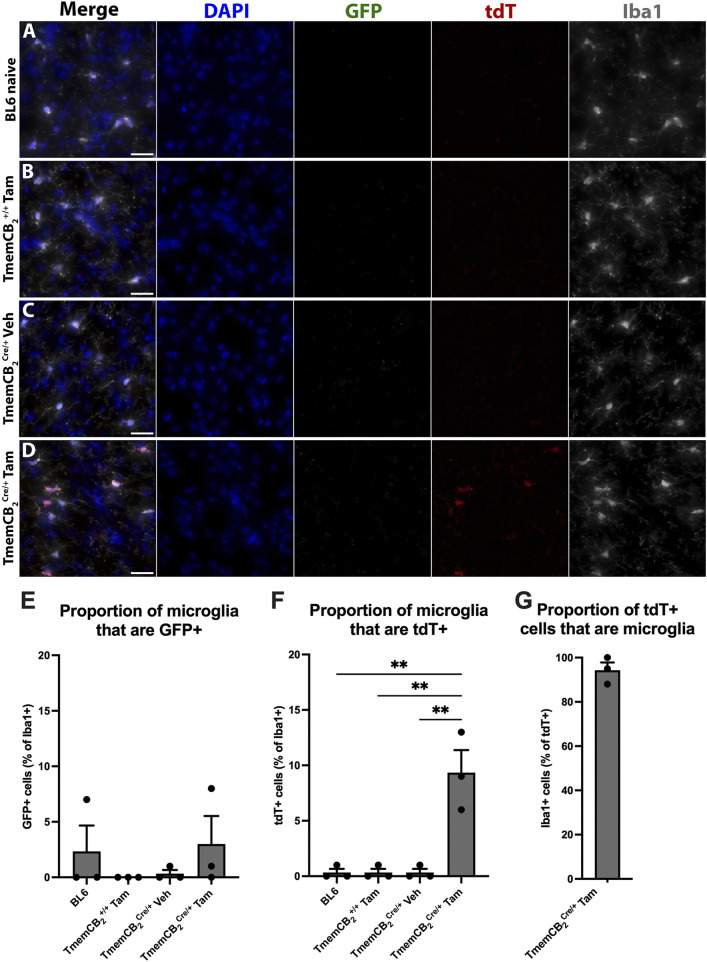
In TmemCB_2_ mice, tdT expression is microglia specific and is Tam/Cre dependent. **(A–D)** Representative images of substantia nigra GFP/tdT/Iba1 immunostaining. **(A)** BL6, **(B)** Tam treated TmemCB_2_^+/+^, **(C)** Vehicle treated TmemCB_2_^Cre/+^ and **(D)** Tam treated TmemCB_2_^Cre/+^. Scale bar = 50 µm. **(E–G)** Quantification of GFP and tdT with Iba1+ microglia. As only one group expressed tdT, G contains one panel only. N = 3/group. An average of 115.8 (±6.1) Iba1+ cells were counted per n. Data are represented as mean ± SEM. One-way ANOVA, Tukey’s *post hoc* test **p ≤ 0.01.

Reporter gene expression and colocalization with Iba1 were analysed to determine KO efficiency (representative images in Figures 7A–D). GFP expression in Iba1+ microglia did not differ between TmemCB_2_ groups and BL6 controls, suggesting a lack of basal CB_2_ expression in microglia under naive conditions (*F* (3, 8) = 0.732, *p* = 0.561; Figure 7E). Similarly, tdT expression in TmemCB_2_^+/+^ and vehicle-treated controls did not differ from BL6 mice, confirming an absence of CB_2_-KO cells. However, in Tam-treated TmemCB_2_^Cre/+^ mice, 9.3% of microglia expressed tdT, indicating a significant increase in CB_2_-KO microglia (*F* (3, 8) = 18.23, *p* = 0.0006; Figure 7F). This confirms that tdT expression in microglia is Tam-dependent. Furthermore, in this group, 94.3% of tdT signal colocalized with Iba1+ microglia, demonstrating that CB_2_-KO is microglia-specific in the CNS (Figure 7G). Low magnification representative images further demonstrate the differences in reporter gene expression between groups (Supplementary Figure S7).

**FIGURE 8:**
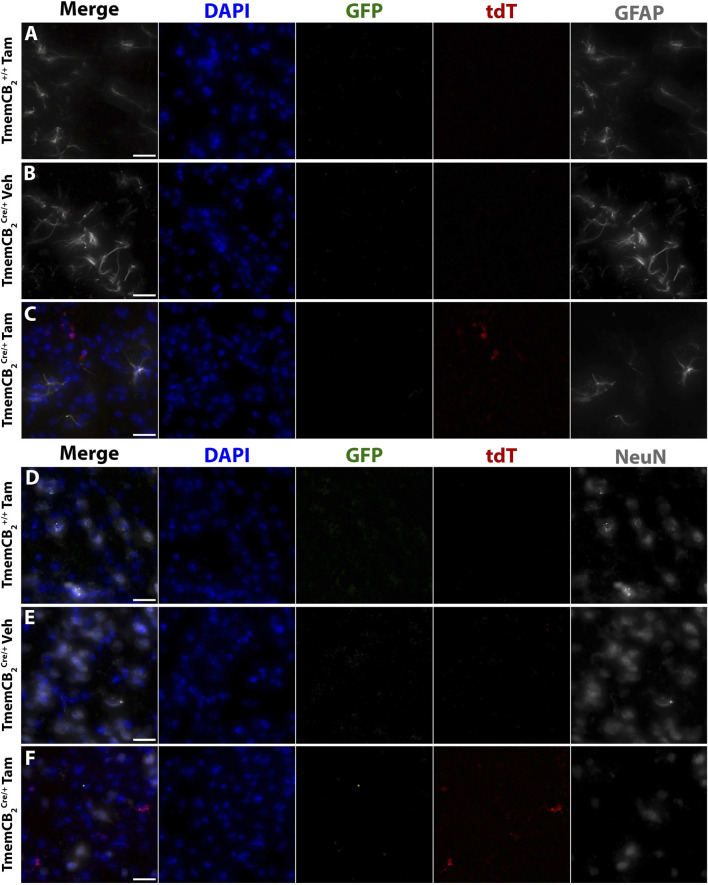
In TmemCB_2_ mice, reporter gene expression does not colocalize with astrocytes or neurons. Representative images of immunostaining in the substantia nigra. GFP and tdT with either **(A–C)** GFAP or **(D–F)** NeuN. **(A,D)** Tam treated TmemCB_2_^+/+^, **(B,E)** Vehicle treated TmemCB_2_^Cre/+^ **(C,F)** Tam treated TmemCB_2_^Cre/+^. Scale bar = 20 µm.

Neither GFP nor tdT colocalized with astrocytes (Figures 8A–C) or neurons (Figures 8D–F) in any group, further confirming that CB_2_ is not detectably expressed in these CNS types under basal conditions and that CB_2_-KO is microglia-specific.

**FIGURE 9:**
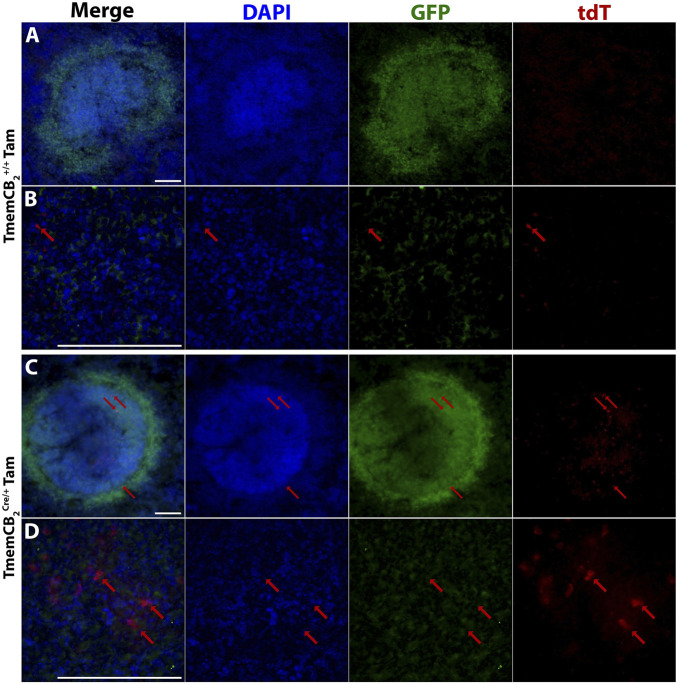
Reporter gene expression is present in the TmemCB_2_ spleen. Representative immunofluorescent images of Tam treated **(A,B)** TmemCB_2_^+/+^ and **(C,D)** TmemCB_2_^Cre/+^ spleens. Red arrows = tdT expression. Scale bar = 100 µm.

Finally, we examined reporter gene expression in the periphery of Tam- or vehicle-treated TmemCB_2_ mice. As expected, minimal tdT expression was observed in the spleen of Tam-treated TmemCB_2_^+/+^ mice (Figures 9A,B). However, rare tdT expression was detected in the spleen of Tam-treated TmemCB_2_^Cre/+^ mice (Figures 9C,D), though at significantly lower levels than in Cx3CB_2_ mice. This suggests that the TmemCB_2_ line exhibits greater specificity for CNS microglia.

### 3.8 Reporter gene expression is upregulated in LPS lesioned mice

To assess whether reporter gene expression accurately reflects CB_2_ upregulation in an inflammatory environment, we examined Tam- and vehicle-treated TmemCB_2_^Cre/+^ mice following LPS-induced neuroinflammation. GFP and tdT expression were analysed in the SN after an ipsilateral intrastriatal injection of LPS to evaluate CB_2_ expression and KO persistence. CD68 is expressed by phagocytic macrophages and is considered a marker for activated microglia within the CNS (Walker and Lue, 2015). Since CB_2_ is upregulated in activated microglia, the colocalization of CD68 with GFP and tdT was assessed to determine the extent of CB_2_ expression in pro-inflammatory microglia in the striatum of this model.

**FIGURE 10:**
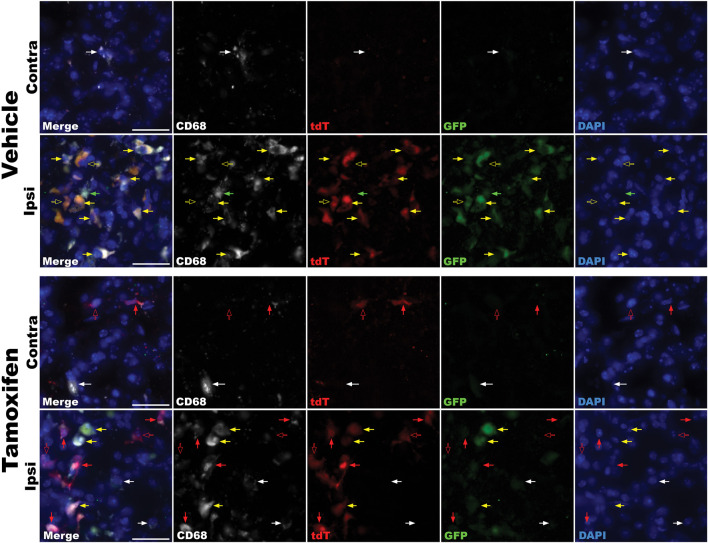
Immunofluorescence analysis of reporter gene expression in the striatum of LPS-lesioned TmemCB_2_^Cre/+^ mice. Representative images from vehicle- and tamoxifen-treated mice are shown for both ipsilateral and contralateral striata. Red arrows = tdT expression. Green arrows = GFP expression. Yellow arrows = GFP and tdT coexpression. Solid arrows = reporter gene colocalizing with CD68. Hollow arrows = reporter gene not colocalizing with CD68. White arrows indicate CD68 expression only. Scale bar = 25uM.

In both treatment groups, GFP expression was highly upregulated in the ipsilateral striatum, indicating a robust increase in CB_2_ expression in response to LPS. GFP almost always colocalized with CD68+ cells, which mark activated microglia, although rare GFP+ CD68− cells were observed (Figure 10).

In vehicle-treated mice, tdT expression was faint and restricted to the lesion site. This tdT signal exclusively colocalized with areas of intense GFP immunoreactivity, suggesting that it arose from channel bleed-through rather than true tdT expression (Figure 10).

In Tam-treated mice, tdT expression was widespread, observed throughout the brain and most prominently at the lesion site. Bright tdT signals were found in the ipsilateral and contralateral striatum, not colocalizing with GFP, indicating true signal and KO of CB_2_ in these cells. In the ipsilateral striatum, tdT predominantly, but not exclusively, colocalized with CD68 (Figure 10).

These findings demonstrate that neuroinflammation induces CB_2_ upregulation, as indicated by increased ipsilateral reporter gene expression in both groups. Importantly, Tam-treated mice exhibited persistent and specific CB_2_-KO throughout the brain under inflammatory conditions.

## 4 Discussion

This study successfully developed and validated a novel microglia-specific, inducible CB_2_-KO mouse model that incorporates a dual reporter system to enable precise, simultaneous visualisation of CB_2_ expression and gene deletion. While floxed *Cnr2* mouse lines (Q.-R. Liu et al., 2017; Stempel et al., 2016) and CB_2_-reporter lines (López et al., 2018; Schmöle et al., 2015) have been generated previously, this is the first model to combine an inducible CB_2_-KO with reporter genes. This unique configuration allows both tracking of endogenous CB_2_ promoter activity and permanent labelling of cells that have undergone CB_2_-KO, a feature not present in any previously described CB_2_-KO models. The CB_2_flx line can be crossed with any Cre driver line, providing a versatile tool to investigate CB_2_ function and regulation in both the CNS and the periphery. This flexibility represents a significant advancement for CB_2_ research.

Given the well-documented lack of reliable CB_2_-specific antibodies (Atwood and Mackie, 2010; Grabon et al., 2023b; Eraso-Pichot et al., 2023), traditional biochemical and histological methods for detecting CB_2_ expression remain limited. By placing the reporter genes under the endogenous *Cnr2* promoter, our approach overcomes this critical methodological barrier: GFP fluorescence enables identification of cells actively expressing CB_2_, while tdT provides a permanent genetic marker of recombination. This allows the monitoring of *Cnr2* promoter activity even after CB_2_ protein has been knocked out, providing unique insights into transcriptional regulation that are not possible with antibody-based methods. Moreover, this design enables assessment of how CB_2_ deletion in 1 cell type may influence CB_2_ expression in other cell populations, supporting more nuanced investigation of intercellular signalling dynamics. While alternative methods such as RNAscope *in situ* hybridisation can detect low-abundance CB_2_ mRNA with high sensitivity, including in neuronal populations where CB_2_ expression is minimal (Eraso-Pichot et al., 2023), our model offers complementary advantages for long-term *in vivo* tracking and functional manipulation of CB_2_-expressing cells without requiring tissue processing or probe optimisation for each experimental condition. However, we do note the limitations of reporter gene lines in detecting low abundance CB_2_ expression.

CB_2_ is known to play critical roles in reproduction, development, and motor control (Atwood and Mackie, 2010), so it was essential to confirm that genetic modifications made to the *Cnr2* gene did not disrupt these functions before microglial KO was induced. Our findings demonstrate these physiological processes remain intact. Additionally, *in vitro* studies revealed no differences in the responses of genetically modified glia to a CB_2_ agonist, indicating that CB_2_ signalling remained intact prior to conditional KO.

Under basal conditions, GFP expression, marking CB_2_ activity, was detected in peripheral tissues but was minimal in the CNS, consistent with previous GFP-CB_2_ models (López et al., 2018; Ruiz De Martín Esteban et al., 2022). Following LPS-induced neuroinflammation, GFP expression increased in microglia, confirming its association with microglial activation and mirroring results from previous research, including those using CB_2_-GFP mice in disease models (López et al., 2018). These findings validate the responsiveness and fidelity of the *Cnr2* promoter in driving reporter expression under neuroinflammatory conditions. Furthermore, Tam-induced Cre activity in the TmemCB_2_ and Cx3CB_2_ lines successfully triggered expression of tdT, indicating successful CB_2_-KO. Importantly, tdT expression was exclusively observed in microglia of Cre/+ mice following Tam administration, validating the inducibility and specificity of the Cre-lox system.

Under inflammatory conditions, reporter gene expression was upregulated in microglia, confirming CB_2_ upregulation, consistent with previous reports (Grabon et al., 2023b). While CB_2_ primarily co-localised with pro-inflammatory microglia, distinct populations were observed, suggesting that CB_2_ expression is heterogeneous among microglial subtypes. The diversity of microglia expression profiles and how this translates to differences in phenotype and function is only beginning to be understood (Paolicelli et al., 2022), so it is not surprising that not all activated microglia ubiquitously express CB_2_ and CD68. Another explanation is that CD68+CB_2_+ cells at the LPS lesion site could be non-microglial cells expressing CB_2_, but given that no GFP expression was observed in neurons or astrocytes, this is unlikely. Future studies incorporating additional markers of reactive microglia could provide further insight into microglial heterogeneity and CB_2_ expression. Additionally, investigation into reporter gene colocalization with markers for other CNS cell types under various physiological and pathological conditions may reveal context-specific upregulation of CB_2_ in these populations. Although there is little evidence for CB_2_ expression in other CNS cell types, such as oligodendrocytes, future studies may wish to validate reporter gene expression in other cell types (Bernal-Chico et al., 2023). One limitation of this model in assessing CB_2_-KO efficiency is that tdT, the marker of successful CB_2_-KO, is only expressed if the *Cnr2* promoter is active. Since basal CB_2_ expression is very low in the CNS, most cells with CB_2_-KO would not be expected to express tdT. Nonetheless, our findings confirm that at least 9.3% of microglia in the TmemCB_2_ line and 91.7% in the Cx3CB_2_ line exhibited CB_2_-KO. Additionally, it is important to note that almost all microglia in the Cx3CB_2_ line and almost all reporter gene expressing microglia in the TmemCB_2_ line expressed tdT, indicating high KO efficiency. To address challenges posed by low basal CB_2_ expression, DNA-based methods such as *in situ* hybridisation will be essential for definitive confirmation of CB_2_-KO efficiency.

Expression levels of tdT in Cre+ animals receiving Tam were significantly higher than GFP expression in control groups, despite both reporters being driven by the *Cnr2* promoter. This discrepancy suggests that *Cnr2* promoter activity is increased following Cre-mediated recombination, complicating the accurate assessment of CB_2_-KO efficiency through reporter genes. This is unlikely to be an effect of Tam, as Tam treated +/+ groups did not have increased *Cnr2* promoter activity, as indicated by GFP expression (Figures 6A, 7E).

A potential explanation for both the increased tdT expression compared to GFP expression in controls and the higher tdT expression in the Cx3CB_2_ line compared to the TmemCB_2_ line may be Cre toxicity. Cre recombinase, after Tam-induced translocation to the nucleus, has been shown to cause DNA damage or other toxic effects that alter cellular function (Sahasrabuddhe and Ghosh, 2022), potentially resulting in increased *Cnr2* promoter activity. Notably, this effect appears to be more pronounced in the Cx3CB_2_ line, where tdT expression was nearly tenfold higher than in the TmemCB_2_ line. This aligns with previous reports suggesting that Cre toxicity is more significant in Cx3cr1-Cre mice compared to Tmem119-Cre lines, possibly due to differences in Cre expression levels (Sahasrabuddhe and Ghosh, 2022). These findings highlight the importance of considering Cre-mediated effects when interpreting data from Cre-lox models, particularly in systems where promoter activity is under investigation; however, further research is required to validate this hypothesis.

Analysis of tdT expression in peripheral tissues also highlighted limitations in the specificity of the Cre drivers used. In the Cx3CB_2_ line, tdT expression persisted in the spleen 3 weeks post-Tam, despite the expectation that Cre-mediated recombination would be restricted to CNS microglia due to rapid turnover of peripheral Cx3cr1+ cells after this time. This persistence suggests that a longer washout period may be necessary to achieve microglia-specific recombination in this line. In contrast, tdT expression in TmemCB_2_ spleens was minimal, supporting the evidence that Tmem119 provides greater specificity to microglia. However, our findings also contribute to emerging evidence that Tmem119 is expressed at low levels outside the CNS. Additionally, given that Tmem119 is downregulated in activated microglia, further investigation is needed to determine its specificity and sensitivity as a microglial marker under both homeostatic and inflammatory conditions (Vankriekelsvenne et al., 2022; Bedolla et al., 2024).

Although most studies using Cx3cr1^Cre/ERT2^ lines to target microglia suggest that a 3-week washout period will result in microglia specificity (Bedolla et al., 2023; Costa et al., 2021; Peng et al., 2016; Mo et al., 2019; Hohsfield et al., 2021; Y. Yang et al., 2023), evidence confirming the clearance of peripheral cells with Cre-mediated recombination at this time point is lacking. The assumption that a 3-week washout period is sufficient for peripheral clearance of KO cells is based on studies using neonate (Parkhurst et al., 2013) or adolescent (Goldmann et al., 2013) mice with low-dose Tam administration protocols. As the majority of studies employing Cx3cr1^Cre/ERT2^ lines use adult mice with longer and higher-dose Tam regimens, the persistence of Cre-mediated modification in peripheral macrophages under these conditions remains unclear, and more recent evidence supports our finding that a washout period is not sufficient (Bedolla et al., 2024). Therefore, if microglia-specific CB_2_-KO is required in the Cx3CB_2_ line, a time course study of peripheral tdT expression is needed to determine the minimum washout period required to achieve specificity, as we have confirmed here that 3 weeks is insufficient.

In summary, this study presents the development and validation of a unique CB_2_ transgenic model that integrates inducible KO with dual fluorescent reporters, offering a versatile and powerful tool to investigate CB_2_ function across tissues, disease states, and developmental stages. The flexibility of the CB_2_flx line, which can be crossed with any Cre line, allows for the study of CB_2_ function and expression across various cell types and tissues. This versatility makes the CB_2_flx line an invaluable tool for exploring CB_2_ in a range of contexts, from neuroinflammation to other disease models in which CB_2_ has been suggested to play a role. By directly addressing existing methodological limitations in CB_2_ research, this model enables experiments that were previously not possible, laying the groundwork for future studies aimed at understanding CB_2_’s contribution to disease processes and assessing the therapeutic potential of CB_2_ modulation.

## Data Availability

The original contributions presented in the study are publicly available. This data can be found here: Genbank database, accession number PRJNA1356642.

## Supplementary material

SUPPLEMENTARY FIGURE S1 Floxed Cnr2 construct. Modified Cnr2 gene **(A)** before and **(B)** after Cre mediated recombination. Blue boxes indicate exons. SA = Splice acceptor. pA = poly **(A)**. IRES = internal ribosome entry site. eGFP = enhanced green flouresent protein. UTR = untranslated region.

SUPPLEMENTARY FIGURE S2 Optimisation of cAMP assay for primary glia cultures. Forskolin concentration-response curves were generated for different cell densities to determine optimum cell density and forskolin concentration.

SUPPLEMENTARY FIGURE S3 Sequencing of floxed *Cnr2* gene.

SUPPLEMENTARY FIGURE S4 Representative, low magnification images of Cx3CB_2_ substantia nigra **(A)** Tam treated Cx3CB_2_^+/+^, **(B)** Vehicle treated Cx3CB_2_^Cre/+^, **(C)** Tam treated Cx3CB_2_^Cre/+^. Scale bar = 100 µm.

SUPPLEMENTARY FIGURE S5 In Cx3CB_2_ mice, reporter gene expression does not colocalize with astrocytes or neurons. Representative images of immunostaining in the substantia nigra of GFP and tdT with either **(A–C)** GFAP or **(D–F)** NeuN. **(A,D)** Tam treated Cx3CB_2_^+/+^, **(B,E)** Vehicle treated Cx3CB_2_^Cre/+^, **(C,F)** Tam treated Cx3CB_2_^Cre/+^. Scale bar = 50 µm.

SUPPLEMENTARY FIGURE S6 Reporter gene expression is present in the Cx3CB_2_ spleen. Representative images of spleen immunofluorescence for **(A)** Cx3CB_2_^+/+^ Tam treated, 1 week-post Tam. **(B)** Cx3CB_2_^Cre/+^ Tam treated, 1 week-post Tam. **(C)** Cx3CB_2_^Cre/+^ Tam treated, 3 weeks-post Tam. Scale bar = 100 µm.

SUPPLEMENTARY FIGURE S7 Low magnification representative images of TmemCB_2_ substantia nigra **(A)** Tam treated TmemCB_2_^+/+^, **(B)** Vehicle treated TmemCB_2_^Cre/+^, **(C)** Tam treated TmemCB_2_^Cre/+^. Arrows indicate colocalization of reporter gene with Iba1. Scale bar = 100 µm.

## References

[B1] Alenabi A. Malekinejad H. (2021). Cannabinoids pharmacological effects are beyond the palliative effects: CB2 cannabinoid receptor agonist induced cytotoxicity and apoptosis in human colorectal cancer cells (HT-29). Mol. Cell Biochem. 476 (9), 3285–3301. 10.1007/s11010-021-04158-6 33886060

[B2] Andersen M. S. Bandres-Ciga S. Reynolds R. H. Hardy J. Ryten M. Krohn L. (2021). Heritability enrichment implicates microglia in parkinson's disease pathogenesis. Ann. Neurol. 89 (5), 942–951. 10.1002/ana.26032 33502028 PMC9017316

[B3] Angelova D. M. Brown D. R. (2019). Microglia and the aging brain: are senescent microglia the key to neurodegeneration? J. Neurochem. 151 (6), 676–688. 10.1111/jnc.14860 31478208

[B4] Antignano I. Liu Y. Offermann N. Capasso M. (2023). Aging microglia. Cell. Mol. Life Sci. 80 (5), 126. 10.1007/s00018-023-04775-y 37081238 PMC10119228

[B5] Araki T. Ikegaya Y. Koyama R. (2021). The effects of microglia‐ and astrocyte‐derived factors on neurogenesis in health and disease. Eur. J. Neurosci. 54 (5), 5880–5901. 10.1111/ejn.14969 32920880 PMC8451940

[B6] Arvanitaki E. S. Goulielmaki E. Gkirtzimanaki K. Niotis G. Tsakani E. Nenedaki E. (2024). Microglia-derived extracellular vesicles trigger age-related neurodegeneration upon DNA damage. Proc. Natl. Acad. Sci. U. S. A. 121 (17), e2317402121. 10.1073/pnas.2317402121 38635632 PMC11047102

[B7] Aso E. Juvés S. Maldonado R. Ferrer I. (2013). CB2 cannabinoid receptor agonist ameliorates alzheimer-like phenotype in AβPP/PS1 mice. J. Alzheimer's Dis. 35 (4), 847–858. 10.3233/JAD-130137 23515018

[B8] Atwood B. K. Mackie K. (2010). CB2: a cannabinoid receptor with an identity crisis. Br. J. Pharmacol. 160 (3), 467–479. 10.1111/j.1476-5381.2010.00729.x 20590558 PMC2931549

[B9] Bedolla A. McKinsey G. Ware K. Santander N. Arnold T. Luo Y. (2023). Finding the right tool: a comprehensive evaluation of microglial inducible cre mouse models. bioRxiv. 10.1101/2023.04.17.536878 37131606 PMC10153116

[B10] Bedolla A. Wegman E. Weed M. Stevens M. K. Ware K. Paranjpe A. (2024). Adult microglial TGFβ1 is required for microglia homeostasis *via* an autocrine mechanism to maintain cognitive function in mice. Nat. Commun. 15 (1), 5306. 10.1038/s41467-024-49596-0 38906887 PMC11192737

[B11] Bernal-Chico A. Tepavcevic V. Manterola A. Utrilla C. Matute C. Mato S. (2023). Endocannabinoid signaling in brain diseases: emerging relevance of glial cells. Glia 71 (1), 103–126. 10.1002/glia.24172 35353392 PMC9790551

[B12] Bido S. Muggeo S. Massimino L. Marzi M. J. Giannelli S. G. Melacini E. (2021). Microglia-specific overexpression of alpha-synuclein leads to severe dopaminergic neurodegeneration by phagocytic exhaustion and oxidative toxicity. Nat. Commun. 12 (1), 6237. 10.1038/s41467-021-26519-x 34716339 PMC8556263

[B13] Birkle T. J. Y. Brown G. C. (2023). Syk inhibitors protect against microglia-mediated neuronal loss in culture. Front. Aging Neurosci. 15, 1120952. 10.3389/fnagi.2023.1120952 37009452 PMC10050448

[B14] Borst K. Dumas A. A. Prinz M. (2021). Microglia: immune and non-immune functions. Immunity 54 (10), 2194–2208. 10.1016/j.immuni.2021.09.014 34644556

[B15] Braun M. Khan Z. T. Khan M. B. Kumar M. Ward A. Achyut B. R. (2018). Selective activation of cannabinoid receptor-2 reduces neuroinflammation after traumatic brain injury *via* alternative macrophage polarization. Brain, Behav. Immun. 68, 224–237. 10.1016/j.bbi.2017.10.021 29079445 PMC5767553

[B16] Brusco A. Tagliaferro P. A. Saez T. Onaivi E. S. (2008). Ultrastructural localization of neuronal brain CB2 cannabinoid receptors. Ann. N. Y. Acad. Sci. 1139 (1), 450–457. 10.1196/annals.1432.037 18991892

[B17] Butler C. A. Popescu A. S. Kitchener E. J. A. Allendorf D. H. Puigdellivol M. Brown G. C. (2021). Microglial phagocytosis of neurons in neurodegeneration, and its regulation. J. Neurochem. 158 (3), 621–639. 10.1111/jnc.15327 33608912

[B18] Chadarevian J. P. Hasselmann J. Lahian A. Capocchi J. K. Escobar A. Lim T. E. (2024). Therapeutic potential of human microglia transplantation in a chimeric model of CSF1R-related leukoencephalopathy. Neuron 112 (16), 2686–2707 e8. 10.1016/j.neuron.2024.05.023 38897209 PMC12357648

[B19] Chen X. Firulyova M. Manis M. Herz J. Smirnov I. Aladyeva E. (2023). Microglia-mediated T cell infiltration drives neurodegeneration in tauopathy. Nature 615 (7953), 668–677. 10.1038/s41586-023-05788-0 36890231 PMC10258627

[B20] Chen S. X. Li Z. Yang L. Xu Z. J. Liu A. P. He Q. W. (2025). Cannabinoid Receptor-2 alleviates sepsis-induced neuroinflammation by modulating microglia M1/M2 subset polarization through inhibiting Nogo-B expression. Mol. Neurobiol. 62, 9258–9270. 10.1007/s12035-025-04836-2 40102346

[B21] Chung Y. C. Shin W.-Ho Baek J. Y. Cho E. J. Baik H. H. Kim S. R. (2016). CB2 receptor activation prevents glial-derived neurotoxic mediator production, BBB leakage and peripheral immune cell infiltration and rescues dopamine neurons in the MPTP model of Parkinson’s disease. Exp. and Mol. Med. 48 (1), e205. 10.1038/emm.2015.100 27534533 PMC4892852

[B22] Consortium, International Multiple Sclerosis Genetics (2019). Multiple sclerosis genomic map implicates peripheral immune cells and microglia in susceptibility. Science 365 (6460), eaav7188. 10.1126/science.aav7188 31604244 PMC7241648

[B23] Corley E. Holleran L. Fahey L. Corvin A. Morris D. W. Donohoe G. (2021). Microglial-expressed genetic risk variants, cognitive function and brain volume in patients with schizophrenia and healthy controls. Transl. Psychiatry 11 (1), 490. 10.1038/s41398-021-01616-z 34556640 PMC8460789

[B24] Costa A. Haage V. Yang S. Wegner S. Ersoy B. Ugursu B. (2021). Deletion of muscarinic acetylcholine receptor 3 in microglia impacts brain ischemic injury. Brain Behav. Immun. 91, 89–104. 10.1016/j.bbi.2020.09.008 32927021

[B25] Cui F. Xu Z. Lv Y. Hu J. (2021). Role of spindle pole body component 25 in neurodegeneration. Ann. Transl. Med. 9 (18), 1432. 10.21037/atm-21-4064 34733984 PMC8506722

[B26] Daria A. Colombo A. Llovera G. Hampel H. Willem M. Liesz A. (2017). Young microglia restore amyloid plaque clearance of aged microglia. EMBO J. 36 (5), 583–603. 10.15252/embj.201694591 28007893 PMC5331757

[B27] Della Valle I. Milani M. Rossi S. Turchi R. Tortolici F. Nesci V. (2024). Loss of homeostatic functions in microglia from a murine model of friedreich's ataxia. Genes Dis. 11 (6), 101178. 10.1016/j.gendis.2023.101178 39100202 PMC11295442

[B28] Deng I. Corrigan F. Zhai G. Zhou X. F. Bobrovskaya L. (2020). Lipopolysaccharide animal models of parkinson's disease: recent progress and relevance to clinical disease. Brain Behav. Immun. Health 4, 100060. 10.1016/j.bbih.2020.100060 34589845 PMC8474547

[B29] Di Marzo V. (2018). New approaches and challenges to targeting the endocannabinoid system. Nat. Rev. Drug Discov. 17 (9), 623–639. 10.1038/nrd.2018.115 30116049

[B30] Dick S. A. Macklin J. A. Nejat S. Momen A. Clemente-Casares X. Althagafi M. G. (2019). Self-renewing resident cardiac macrophages limit adverse remodeling following myocardial infarction. Nat. Immunol. 20 (1), 29–39. 10.1038/s41590-018-0272-2 30538339 PMC6565365

[B31] Ding X. Wang J. Huang M. Chen Z. Liu J. Zhang Q. (2021). Loss of microglial SIRPα promotes synaptic pruning in preclinical models of neurodegeneration. Nat. Commun. 12 (1), 2030. 10.1038/s41467-021-22301-1 33795678 PMC8016980

[B32] Dong Y. D'Mello C. Pinsky W. Lozinski B. M. Kaushik D. K. Ghorbani S. (2021). Oxidized phosphatidylcholines found in multiple sclerosis lesions mediate neurodegeneration and are neutralized by microglia. Nat. Neurosci. 24 (4), 489–503. 10.1038/s41593-021-00801-z 33603230

[B33] Duffy S. S. Hayes J. P. Fiore N. T. Moalem-Taylor G. (2021). The cannabinoid system and microglia in health and disease. Neuropharmacology 190, 108555. 10.1016/j.neuropharm.2021.108555 33845074

[B34] Eraso-Pichot A. Pouvreau S. Olivera-Pinto A. Gomez-Sotres P. Skupio U. Marsicano G. (2023). Endocannabinoid signaling in astrocytes. Glia 71 (1), 44–59. 10.1002/glia.24246 35822691 PMC9796923

[B35] Espadas I. Keifman E. Palomo-Garo C. Burgaz S. García C. Fernández-Ruiz J. (2020). Beneficial effects of the phytocannabinoid Δ9-THCV in L-DOPA-induced dyskinesia in parkinson's disease. Neurobiol. Dis. 141, 104892. 10.1016/j.nbd.2020.104892 32387338

[B36] Espejo-Porras F. Toscano L. G. Cueto C. R. Santos-García I. Lago E.De Ruiz J. F. (2019). Targeting glial cannabinoid CB_2_ receptors to delay the progression of the pathological phenotype in TDP-43 (A315T) transgenic mice, a model of amyotrophic lateral sclerosis. Br. J. Pharmacol. 176 (10), 1585–1600. 10.1111/bph.14216 29574689 PMC6487601

[B37] Fakhfouri G. Ahmadiani A. Rahimian R. Grolla A. A. Moradi F. Ali H. (2012). WIN55212-2 attenuates amyloid-beta-induced neuroinflammation in rats through activation of cannabinoid receptors and PPAR-γ pathway. Neuropharmacology 63 (4), 653–666. 10.1016/j.neuropharm.2012.05.013 22634229

[B38] Ferranti A. S. Foster D. J. (2022). Cannabinoid type-2 receptors: an emerging target for regulating schizophrenia-relevant brain circuits. Front. Neurosci. 16, 925792. 10.3389/fnins.2022.925792 36033626 PMC9403189

[B39] Fricker M. Oliva-Martin M. J. Brown G. C. (2012). Primary phagocytosis of viable neurons by microglia activated with LPS or abeta is dependent on calreticulin/LRP phagocytic signalling. J. Neuroinflammation 9, 196. 10.1186/1742-2094-9-196 22889139 PMC3481398

[B40] Galiègue S. Mary S. Marchand J. Dussossoy D. Carrière D. Carayon P. (1995). Expression of central and peripheral cannabinoid receptors in human immune tissues and leukocyte subpopulations. Eur. J. Biochem. 232 (1), 54–61. 10.1111/j.1432-1033.1995.tb20780.x 7556170

[B41] Gambacorta N. Gasperi V. Guzzo T. Di Leva F. S. Ciriaco F. Sanchez C. (2023). Exploring the 1,3-benzoxazine chemotype for cannabinoid receptor 2 as a promising anti-cancer therapeutic. Eur. J. Med. Chem. 259, 115647. 10.1016/j.ejmech.2023.115647 37478557

[B42] Gao C. Jiang J. Tan Y. Chen S. (2023). Microglia in neurodegenerative diseases: mechanism and potential therapeutic targets. Signal Transduct. Target Ther. 8 (1), 359. 10.1038/s41392-023-01588-0 37735487 PMC10514343

[B43] Gasperi V. Guzzo T. Topai A. Gambacorta N. Ciriaco F. Nicolotti O. (2023). Recent advances on Type-2 cannabinoid (CB2) receptor agonists and their therapeutic potential. Curr. Med. Chem. 30 (12), 1420–1457. 10.2174/0929867329666220825161603 36028971

[B44] Goldmann T. Wieghofer P. Müller P. F. Wolf Y. Varol D. Yona S. (2013). A new type of microglia gene targeting shows TAK1 to be pivotal in CNS autoimmune inflammation. Nat. Neurosci. 16 (11), 1618–1626. 10.1038/nn.3531 24077561

[B45] Gómez-Gálvez Y. Palomo-Garo C. Fernández-Ruiz J. García C. (2016). Potential of the cannabinoid CB2 receptor as a pharmacological target against inflammation in parkinson's disease. Prog. Neuro-Psychopharmacology Biol. Psychiatry 64, 200–208. 10.1016/j.pnpbp.2015.03.017 25863279

[B46] Grabon W. Bodennec J. Rheims S. Belmeguenai A. Bezin L. (2023a). Update on the controversial identity of cells expressing cnr2 gene in the nervous system. CNS Neurosci. and Ther. 29 (3), 760–770. 10.1111/cns.13977 36604187 PMC9928557

[B47] Grabon W. Rheims S. Smith J. Bodennec J. Belmeguenai A. Bezin L. (2023b). CB2 receptor in the CNS: from immune and neuronal modulation to behavior. Neurosci. and Biobehav. Rev. 150, 105226. 10.1016/j.neubiorev.2023.105226 37164044

[B48] Gratuze M. Schlachetzki J. C. M. D'Oliveira Albanus R. Jain N. Novotny B. Brase L. (2023). TREM2-independent microgliosis promotes tau-mediated neurodegeneration in the presence of ApoE4. Neuron 111 (2), 202–219 e7. 10.1016/j.neuron.2022.10.022 36368315 PMC9852006

[B49] Green G. S. Fujita M. Yang H. S. Taga M. Cain A. McCabe C. (2024). Cellular communities reveal trajectories of brain ageing and alzheimer's disease. Nature 633 (8030), 634–645. 10.1038/s41586-024-07871-6 39198642 PMC11877878

[B50] Guo S. Wang H. Yin Y. (2022). Microglia polarization from M1 to M2 in neurodegenerative diseases. Front. Aging Neurosci. 14, 815347. 10.3389/fnagi.2022.815347 35250543 PMC8888930

[B51] Hohsfield L. A. Najafi A. R. Ghorbanian Y. Soni N. Crapser J. Figueroa Velez D. X. (2021). Subventricular zone/white matter microglia reconstitute the empty adult microglial niche in a dynamic wave. Elife 10, e66738. 10.7554/eLife.66738 34423781 PMC8425950

[B52] Hosoki H. Asahi T. Nozaki C. (2024). Cannabinoid CB2 receptors enhance high-fat diet evoked peripheral neuroinflammation. Life Sci. 355, 123002. 10.1016/j.lfs.2024.123002 39173999

[B53] Jang J. Hong A. Chung Y. Jin B. (2022). Interleukin-4 aggravates LPS-induced striatal neurodegeneration *in vivo via* oxidative stress and polarization of microglia/macrophages. Int. J. Mol. Sci. 23 (1), 571. 10.3390/ijms23010571 35008995 PMC8745503

[B54] Jayant S. Mohan Sharma B. Bansal R. Sharma B. (2016). Pharmacological benefits of selective modulation of cannabinoid receptor type 2 (CB2) in experimental alzheimer's disease. Pharmacol. Biochem. Behav. 140, 39–50. 10.1016/j.pbb.2015.11.006 26577751

[B55] Jia Yi Han D. Qin Q. Ma Z. G. (2020). JWH133 inhibits MPP+-Induced inflammatory response and iron influx in astrocytes. Neurosci. Lett. 720, 134779. 10.1016/j.neulet.2020.134779 31981721

[B56] Jing L. Hou L. Zhang D. Li S. Ruan Z. Zhang X. (2021). Microglial activation mediates noradrenergic locus coeruleus neurodegeneration *via* complement receptor 3 in a rotenone-induced parkinson's disease mouse model. J. Inflamm. Res. 14, 1341–1356. 10.2147/JIR.S299927 33859489 PMC8044341

[B57] Joers V. Murray B. C. McLaughlin C. Oliver D. Staley H. E. Coronado J. (2024). Modulation of cannabinoid receptor 2 alters neuroinflammation and reduces formation of alpha-synuclein aggregates in a rat model of nigral synucleinopathy. J. Neuroinflammation 21 (1), 240. 10.1186/s12974-024-03221-5 39334169 PMC11438102

[B58] Joshi N. Onaivi E. S. (2019). “Endocannabinoid system components: overview and tissue distribution,” in Recent advances in cannabinoid physiology and pathology. Editor Bukiya A. N. (Cham: Springer International Publishing), 1–12. 10.1007/978-3-030-21737-2_1 31332731

[B59] Joshi A. U. Minhas P. S. Liddelow S. A. Haileselassie B. Andreasson K. I. Dorn G. W. 2nd (2019). Fragmented mitochondria released from microglia trigger A1 astrocytic response and propagate inflammatory neurodegeneration. Nat. Neurosci. 22 (10), 1635–1648. 10.1038/s41593-019-0486-0 31551592 PMC6764589

[B60] Kaiser T. Feng G. (2019). Tmem119-EGFP and Tmem119-CreERT2 transgenic mice for labeling and manipulating microglia. eNeuro 6 (4), ENEURO.0448–18.2019. 10.1523/ENEURO.0448-18.2019 31371457 PMC6712208

[B61] Kaminski N. E. (1998). Inhibition of the cAMP signaling Cascade *via* cannabinoid receptors: a putative mechanism of immune modulation by cannabinoid compounds. Toxicol. Lett. 102-103, 59–63. 10.1016/s0378-4274(98)00284-7 10022233

[B62] Kang Y. J. Hyeon S. J. McQuade A. Lim J. Baek S. H. Diep Y. N. (2024). Neurotoxic microglial activation *via* IFNγ-Induced Nrf2 reduction exacerbating alzheimer's disease. Adv. Sci. (Weinh) 11 (20), e2304357. 10.1002/advs.202304357 38482922 PMC11132036

[B63] Kitchener E. J. A. Dundee J. M. Brown G. C. (2023). Activated microglia release beta-galactosidase that promotes inflammatory neurodegeneration. Front. Aging Neurosci. 15, 1327756. 10.3389/fnagi.2023.1327756 38283068 PMC10811154

[B64] Kong W. Li H. Tuma R. F. Ganea D. (2014). Selective CB2 receptor activation ameliorates EAE by reducing Th17 differentiation and immune cell accumulation in the CNS. Cell. Immunol. 287 (1), 1–17. 10.1016/j.cellimm.2013.11.002 24342422 PMC3906668

[B65] Kong W. Xie Z. Shang X. Hayashi Y. Lan F. Zhao S. (2023). Zinc finger protein 335 mediates lipopolysaccharide-induced neurodegeneration and memory loss as a transcriptional factor in microglia. Glia 71 (12), 2720–2734. 10.1002/glia.24447 37522284

[B66] Kwon H. S. Koh S. H. (2020). Neuroinflammation in neurodegenerative disorders: the roles of microglia and astrocytes. Transl. Neurodegener. 9 (1), 42. 10.1186/s40035-020-00221-2 33239064 PMC7689983

[B67] Lee J. Kim D. E. Griffin P. Sheehan P. W. Kim D. H. Musiek E. S. (2020). Inhibition of REV-ERBs stimulates microglial amyloid-beta clearance and reduces amyloid plaque deposition in the 5XFAD mouse model of alzheimer's disease. Aging Cell 19 (2), e13078. 10.1111/acel.13078 31800167 PMC6996949

[B68] Lewis S. M. Williams A. Eisenbarth S. C. (2019). Structure and function of the immune system in the spleen. Sci. Immunol. 4 (33), eaau6085. 10.1126/sciimmunol.aau6085 30824527 PMC6495537

[B69] Li C. Shi J. Wang Bo Jin Li Jia H. (2019). CB2 cannabinoid receptor agonist ameliorates novel object recognition but not spatial memory in transgenic APP/PS1 mice. Neurosci. Lett. 707, 134286. 10.1016/j.neulet.2019.134286 31150731

[B70] Li Q. Shen C. Liu Z. Ma Y. Wang J. Dong H. (2021). Partial depletion and repopulation of microglia have different effects in the acute MPTP mouse model of Parkinson’s disease. Cell Prolif. 54 (8), e13094. 10.1111/cpr.13094 34312932 PMC8349650

[B71] Liang S. Q. Li P. H. Hu Y. Y. Zhao J. L. Shao F. Z. Kuang F. (2023). Myeloid-specific blockade of notch signaling alleviates dopaminergic neurodegeneration in parkinson's disease by dominantly regulating resident microglia activation through NF-κB signaling. Front. Immunol. 14, 1193081. 10.3389/fimmu.2023.1193081 37680624 PMC10481959

[B72] Liddelow S. A. Guttenplan K. A. Clarke L. E. Bennett F. C. Bohlen C. J. Schirmer L. (2017). Neurotoxic reactive astrocytes are induced by activated microglia. Nature 541 (7638), 481–487. 10.1038/nature21029 28099414 PMC5404890

[B73] Lindhout I. A. Murray T. E. Richards C. M. Klegeris A. (2021). Potential neurotoxic activity of diverse molecules released by microglia. Neurochem. Int. 148. 10.1016/j.neuint.2021.105117 34186114

[B74] Liu Q.-R. Canseco-Alba A. Zhang H.-Y. Tagliaferro P. Chung M. Dennis E. (2017). Cannabinoid type 2 receptors in dopamine neurons inhibits psychomotor behaviors, alters anxiety, depression and alcohol preference. Sci. Rep. 7 (1), 17410. 10.1038/s41598-017-17796-y 29234141 PMC5727179

[B75] Liu X. Chen Y. Wang H. Wei Y. Yuan Y. Zhou Q. (2021). Microglia-derived IL-1β promoted neuronal apoptosis through ER stress-mediated signaling pathway PERK/eIF2α/ATF4/CHOP upon arsenic exposure. J. Hazard Mater 417, 125997. 10.1016/j.jhazmat.2021.125997 34229406

[B76] Liu X. Yu H. Chen B. Friedman V. Mu L. Kelly T. J. (2022). CB2 agonist GW842166x protected against 6-OHDA-Induced Anxiogenic- and depressive-related behaviors in mice. Biomedicines 10 (8), 1776. 10.3390/biomedicines10081776 35892676 PMC9329798

[B77] López A. Aparicio N. Ruth Pazos M. Teresa Grande M. Asunción Barreda-Manso M. Benito-Cuesta I. (2018). Cannabinoid CB2 receptors in the mouse brain: relevance for Alzheimer’s disease. J. Neuroinflammation 15 (1), 158. 10.1186/s12974-018-1174-9 29793509 PMC5968596

[B78] Lowe H. Toyang N. Steele B. Bryant J. Ngwa W. (2021). The endocannabinoid system: a potential target for the treatment of various diseases. Int. J. Mol. Sci. 22 (17), 9472. 10.3390/ijms22179472 34502379 PMC8430969

[B79] Lunde A. Glover J. C. (2020). A versatile toolbox for semi-automatic cell-by-cell object-based colocalization analysis. Sci. Rep. 10 (1), 19027. 10.1038/s41598-020-75835-7 33149236 PMC7643144

[B80] Mader M. M. Napole A. Wu D. Atkins M. Scavetti A. Shibuya Y. (2024). Myeloid cell replacement is neuroprotective in chronic experimental autoimmune encephalomyelitis. Nat. Neurosci. 27 (5), 901–912. 10.1038/s41593-024-01609-3 38514857

[B81] Mizutani M. Pino P. A. Saederup N. Charo I. F. Ransohoff R. M. Cardona A. E. (2012). The fractalkine receptor but not CCR2 is present on microglia from embryonic development throughout adulthood. J. Immunol. 188 (1), 29–36. 10.4049/jimmunol.1100421 22079990 PMC3244524

[B82] Mo M. Eyo U. B. Xie M. Peng J. Bosco D. B. Umpierre A. D. (2019). Microglial P2Y12 receptor regulates seizure-induced neurogenesis and immature neuronal projections. J. Neurosci. 39 (47), 9453–9464. 10.1523/jneurosci.0487-19.2019 31597724 PMC6867812

[B83] Monory K. Lutz B. (2005). Pain killer without a high. Nat. Med. 11 (4), 378–379. 10.1038/nm0405-378 15812515

[B84] More S. A. Deore R. S. Pawar H. D. Sharma C. Nakhate K. T. Rathod S. S. (2024). CB2 cannabinoid receptor as a potential target in myocardial infarction: exploration of molecular pathogenesis and therapeutic strategies. Int. J. Mol. Sci. 25 (3), 1683. 10.3390/ijms25031683 38338960 PMC10855244

[B85] Moreno M. Brera B. Spuch C. Carro E. García-García L. Delgado M. (2012). Prolonged oral cannabinoid administration prevents neuroinflammation, lowers β-amyloid levels and improves cognitive performance in Tg APP 2576 mice. J. Neuroinflammation 9 (1), 8. 10.1186/1742-2094-9-8 22248049 PMC3292807

[B86] Munro D. A. D. Bestard-Cuche N. McQuaid C. Chagnot A. Shabestari S. K. Chadarevian J. P. (2024). Microglia protect against age-associated brain pathologies. Neuron 112 (16), 2732–2748 e8. 10.1016/j.neuron.2024.05.018 38897208

[B87] Nan J. Liu J. Lin G. Zhang S. Xia A. Zhou P. (2023). Discovery of 4-(1,2,4-Oxadiazol-5-yl)azepan-2-one derivatives as a new class of cannabinoid type 2 receptor agonists for the treatment of inflammatory pain. J. Med. Chem. 66 (5), 3460–3483. 10.1021/acs.jmedchem.2c01943 36821347

[B88] Odfalk K. F. Bieniek K. F. Hopp S. C. (2022). Microglia: friend and foe in tauopathy. Prog. Neurobiol. 216, 102306. 10.1016/j.pneurobio.2022.102306 35714860 PMC9378545

[B89] Ojha S. Javed H. Azimullah S. Haque M. E. (2016). β-Caryophyllene, a phytocannabinoid attenuates oxidative stress, neuroinflammation, glial activation, and salvages dopaminergic neurons in a rat model of parkinson disease. Mol. Cell Biochem. 418 (1-2), 59–70. 10.1007/s11010-016-2733-y 27316720

[B90] Onaivi E. S. (2006). Neuropsychobiological evidence for the functional presence and expression of cannabinoid CB2 receptors in the brain. Neuropsychobiology 54 (4), 231–246. 10.1159/000100778 17356307

[B91] Oyarce K. Cepeda M. Y. Lagos R. Garrido C. Vega-Letter A. M. Garcia-Robles M. (2022). Neuroprotective and neurotoxic effects of glial-derived exosomes. Front. Cell Neurosci. 16, 920686. 10.3389/fncel.2022.920686 35813501 PMC9257100

[B92] Palpagama T. H. Waldvogel H. J. Faull R. L. M. Kwakowsky A. (2019). The role of microglia and astrocytes in huntington’s disease. Front. Mol. Neurosci. 12, 258. 10.3389/fnmol.2019.00258 31708741 PMC6824292

[B93] Pan L. Cho K. S. Wei X. Xu F. Lennikov A. Hu G. (2023). IGFBPL1 is a master driver of microglia homeostasis and resolution of neuroinflammation in glaucoma and brain tauopathy. Cell Rep. 42 (8), 112889. 10.1016/j.celrep.2023.112889 37527036 PMC10528709

[B94] Paolicelli R. C. Sierra A. Stevens B. Tremblay M.-E. Aguzzi A. Ajami B. (2022). Microglia states and nomenclature: a field at its crossroads. Neuron 110 (21), 3458–3483. 10.1016/j.neuron.2022.10.020 36327895 PMC9999291

[B95] Parkhurst C. N. Yang G. Ninan I. Savas J. N. Yates J. R. 3rd Lafaille J. J. (2013). Microglia promote learning-dependent synapse formation through brain-derived neurotrophic factor. Cell 155 (7), 1596–1609. 10.1016/j.cell.2013.11.030 24360280 PMC4033691

[B96] Peng J. Gu N. Zhou L. Eyo U B. Murugan M. Gan W. B. (2016). Microglia and monocytes synergistically promote the transition from acute to chronic pain after nerve injury. Nat. Commun. 7, 12029. 10.1038/ncomms12029 27349690 PMC4931235

[B97] Penney J. Ralvenius W. T. Loon A. Cerit O. Dileep V. Milo B. (2024). iPSC-derived microglia carrying the TREM2 R47H/+ mutation are proinflammatory and promote synapse loss. Glia 72 (2), 452–469. 10.1002/glia.24485 37969043 PMC10904109

[B98] Piano I. Votta A. Colucci P. Corsi F. Vitolo S. Cerri C. (2023). Anti-inflammatory reprogramming of microglia cells by metabolic modulators to counteract neurodegeneration; a new role for ranolazine. Sci. Rep. 13 (1), 20138. 10.1038/s41598-023-47540-8 37978212 PMC10656419

[B99] Prinz M. Jung S. Priller J. (2019). Microglia biology: one century of evolving concepts. Cell 179 (2), 292–311. 10.1016/j.cell.2019.08.053 31585077

[B100] Rakotoarivelo V. Mayer T. Z. Simard M. Flamand N. Di Marzo V. (2024). The impact of the CB2 cannabinoid receptor in inflammatory diseases: an update. Molecules 29 (14), 3381. 10.3390/molecules29143381 39064959 PMC11279428

[B101] Rentsch P. Stayte S. Egan T. Clark I. Vissel B. (2020). Targeting the cannabinoid receptor CB2 in a mouse model of l-dopa induced dyskinesia. Neurobiol. Dis. 134. 10.1016/j.nbd.2019.104646 31669673

[B102] Rizzi C. Tiberi A. Giustizieri M. Marrone M. C. Gobbo F. Carucci N. M. (2018). NGF steers microglia toward a neuroprotective phenotype. Glia 66 (7), 1395–1416. 10.1002/glia.23312 29473218 PMC6001573

[B103] Robinson R. H. Meissler J. J. Fan X. Yu D. Adler M. W. Eisenstein T. K. (2015). A CB2-Selective cannabinoid suppresses T-Cell activities and increases tregs and IL-10. J. Neuroimmune Pharmacol. 10 (2), 318–332. 10.1007/s11481-015-9611-3 25980325 PMC4528965

[B104] Rocha S. M. Kirkley K. S. Chatterjee D. Aboellail T. A. Smeyne R. J. Tjalkens R. B. (2023). Microglia-specific knock-out of NF-κB/IKK2 increases the accumulation of misfolded α-synuclein through the inhibition of p62/sequestosome-1-dependent autophagy in the rotenone model of parkinson's disease. Glia 71 (9), 2154–2179. 10.1002/glia.24385 37199240 PMC10330367

[B105] Rodero M. Marie Y. Coudert M. Blondet E. Mokhtari K. Rousseau A. (2008). Polymorphism in the microglial cell-mobilizing CX3CR1 gene is associated with survival in patients with glioblastoma. J. Clin. Oncol. 26 (36), 5957–5964. 10.1200/JCO.2008.17.2833 19001328

[B106] Rodriguez-Gomez J. A. Kavanagh E. Engskog-Vlachos P. Engskog M. K. R. Herrera A. J. Espinosa-Oliva A. M. (2020). Microglia: agents of the CNS pro-inflammatory response. Cells 9 (7), 1717. 10.3390/cells9071717 32709045 PMC7407646

[B107] Rohbeck E. Eckel J. Romacho T. (2021). Cannabinoid receptors in metabolic regulation and diabetes. Physiol. (Bethesda) 36 (2), 102–113. 10.1152/physiol.00029.2020 33595385

[B108] Rorato R. Ferreira N. L. Oliveira F. P. Fideles H. J. Camilo T. A. Antunes-Rodrigues J. (2022). Prolonged activation of brain CB2 signaling modulates hypothalamic microgliosis and astrogliosis in high fat diet-fed mice. Int. J. Mol. Sci. 23 (10), 5527. 10.3390/ijms23105527 35628338 PMC9141740

[B109] Rossi F. Bellini G. Luongo L. Manzo I. Tolone S. Tortora C. (2016). Cannabinoid receptor 2 as antiobesity target: inflammation, fat storage, and browning modulation. J. Clin. Endocrinol. Metab. 101 (9), 3469–3478. 10.1210/jc.2015-4381 27294325

[B110] Ruiz De Martín Esteban S. Benito-Cuesta I. Terradillos I. Martínez-Relimpio A. M. Andrea Arnanz M. Ruiz-Pérez G. (2022). Cannabinoid CB2 receptors modulate microglia function and amyloid dynamics in a mouse model of alzheimer’s disease. Front. Pharmacol. 13, 841766. 10.3389/fphar.2022.841766 35645832 PMC9136843

[B111] Ryan S. K. Zelic M. Han Y. Teeple E. Chen L. Sadeghi M. (2023). Microglia ferroptosis is regulated by SEC24B and contributes to neurodegeneration. Nat. Neurosci. 26 (1), 12–26. 10.1038/s41593-022-01221-3 36536241 PMC9829540

[B112] Sagredo O. González S. Aroyo I. Ruth Pazos M. Benito C. Becker I. L. (2009). Cannabinoid CB2 receptor agonists protect the striatum against malonate toxicity: relevance for huntington's disease. Glia 57 (11), 1154–1167. 10.1002/glia.20838 19115380 PMC2706932

[B113] Sahasrabuddhe V. Ghosh H. S. (2022). Cx3Cr1-Cre induction leads to microglial activation and IFN-1 signaling caused by DNA damage in early postnatal brain. Cell Rep. 38 (3), 110252. 10.1016/j.celrep.2021.110252 35045285

[B114] Salvadores N. Moreno-Gonzalez I. Gamez N. Quiroz G. Vegas-Gomez L. Escandon M. (2022). Aβ oligomers trigger necroptosis-mediated neurodegeneration *via* microglia activation in alzheimer's disease. Acta Neuropathol. Commun. 10 (1), 31. 10.1186/s40478-022-01332-9 35264247 PMC8908658

[B115] Schildge S. Bohrer C. Beck K. Schachtrup C. (2013). Isolation and culture of mouse cortical astrocytes. J. Vis. Exp. 71, 50079. 10.3791/50079 23380713 PMC3582677

[B116] Schmöle A.-C. Lundt R. Ternes S. Albayram Ö. Ulas T. Schultze J. L. (2015). Cannabinoid receptor 2 deficiency results in reduced neuroinflammation in an alzheimer's disease mouse model. Neurobiol. Aging 36 (2), 710–719. 10.1016/j.neurobiolaging.2014.09.019 25443294

[B118] Shi Y. Manis M. Long J. Wang K. Sullivan P. M. Remolina Serrano J. (2019). Microglia drive APOE-Dependent neurodegeneration in a tauopathy mouse model. J. Exp. Med. 216 (11), 2546–2561. 10.1084/jem.20190980 31601677 PMC6829593

[B119] Shibuya Y. Kumar K. K. Mader M. M. Yoo Y. Ayala L. A. Zhou M. (2022). Treatment of a genetic brain disease by CNS-Wide microglia replacement. Sci. Transl. Med. 14 (636), eabl9945. 10.1126/scitranslmed.abl9945 35294256 PMC9618306

[B120] Silver R. J. (2019). The endocannabinoid system of animals. Animals 9 (9), 686. 10.3390/ani9090686 31527410 PMC6770351

[B121] Simard M. Rakotoarivelo V. Di Marzo V. Flamand N. (2022). Expression and functions of the CB2 receptor in human leukocytes. Front. Pharmacol. 13, 826400. 10.3389/fphar.2022.826400 35273503 PMC8902156

[B122] Skrzypczak-Wiercioch A. Salat K. (2022). Lipopolysaccharide-induced model of neuroinflammation: mechanisms of action, research application and future directions for its use. Molecules 27 (17), 5481. 10.3390/molecules27175481 36080253 PMC9457753

[B123] Smoum R. Grether U. Karsak M. Vernall A. J. Park F. Hillard C. J. (2022). Editorial: therapeutic potential of the cannabinoid CB2 receptor. Front. Pharmacol. 13, 1039564. 10.3389/fphar.2022.1039564 36278235 PMC9585503

[B124] Sobue A. Komine O. Hara Y. Endo F. Mizoguchi H. Watanabe S. (2021). Microglial gene signature reveals loss of homeostatic microglia associated with neurodegeneration of alzheimer's disease. Acta Neuropathol. Commun. 9 (1), 1. 10.1186/s40478-020-01099-x 33402227 PMC7786928

[B125] Sobue A. Komine O. Endo F. Kakimi C. Miyoshi Y. Kawade N. (2024). Microglial cannabinoid receptor type II stimulation improves cognitive impairment and neuroinflammation in alzheimer's disease mice by controlling astrocyte activation. Cell Death Dis. 15 (11), 858. 10.1038/s41419-024-07249-6 39587077 PMC11589152

[B126] Stempel A. V. Vanessa A. Stumpf A. Zhang H.-Y. Özdoğan T. Pannasch U. (2016). Cannabinoid type 2 receptors mediate a cell type-specific plasticity in the hippocampus. Neuron 90 (4), 795–809. 10.1016/j.neuron.2016.03.034 27133464 PMC5533103

[B127] Subbarayan M. S. Joly-Amado A. Bickford P. C. Nash K. R. (2022). CX3CL1/CX3CR1 signaling targets for the treatment of neurodegenerative diseases. Pharmacol. Ther. 231, 107989. 10.1016/j.pharmthera.2021.107989 34492237

[B128] Takatori S. Wang W. Iguchi A. Tomita T. (2019). Genetic risk factors for alzheimer disease: emerging roles of microglia in disease pathomechanisms. Adv. Exp. Med. Biol. 1118, 83–116. 10.1007/978-3-030-05542-4_5 30747419

[B129] Tang J. Chen Q. Guo J. Yang L. Tao Y. Lin Li (2016). Minocycline attenuates neonatal germinal-matrix-hemorrhage-induced neuroinflammation and brain edema by activating cannabinoid receptor 2. Mol. Neurobiol. 53 (3), 1935–1948. 10.1007/s12035-015-9154-x 25833102

[B130] Tao L. Liu Q. Zhang F. Fu Y. Zhu X. Weng X. (2021). Microglia modulation with 1070-nm light attenuates Aβ burden and cognitive impairment in alzheimer's disease mouse model. Light Sci. Appl. 10 (1), 179. 10.1038/s41377-021-00617-3 34493703 PMC8423759

[B131] Umpierre A. D. Wu L. J. (2021). How microglia sense and regulate neuronal activity. Glia 69 (7), 1637–1653. 10.1002/glia.23961 33369790 PMC8113084

[B132] van den Hoogen N. J. Harding E. K. Davidson C. E. D. Trang T. (2021). Cannabinoids in chronic pain: therapeutic potential through microglia modulation. Front. Neural Circuits 15, 816747. 10.3389/fncir.2021.816747 35069129 PMC8777271

[B133] Van E. Straub V. M. Stelt M. V. D. (2021). Targeting endocannabinoid signaling: FAAH and MAG lipase inhibitors. Annu. Rev. Pharmacol. Toxicol. 61 (1), 441–463. 10.1146/annurev-pharmtox-030220-112741 32867595

[B134] Vankriekelsvenne E. Chrzanowski U. Manzhula K. Greiner T. Wree A. Hawlitschka A. (2022). Transmembrane protein 119 is neither a specific nor a reliable marker for microglia. Glia 70 (6), 1170–1190. 10.1002/glia.24164 35246882

[B135] Verty A. N. Stefanidis A. McAinch A. J. Hryciw D. H. Oldfield B. (2015). Anti-obesity effect of the CB2 receptor agonist JWH-015 in diet-induced Obese mice. PLoS One 10 (11), e0140592. 10.1371/journal.pone.0140592 26588700 PMC4654496

[B136] Viveros-Paredes J. González-Castañeda R. Gertsch J. Chaparro-Huerta V. López-Roa R. Vázquez-Valls E. (2017). Neuroprotective effects of β-Caryophyllene against dopaminergic neuron injury in a murine model of parkinson’s disease induced by MPTP. Pharmaceuticals 10 (3), 60. 10.3390/ph10030060 28684694 PMC5620604

[B137] Walker D. G. Lue L. F. (2015). Immune phenotypes of microglia in human neurodegenerative disease: challenges to detecting microglial polarization in human brains. Alzheimers Res. Ther. 7 (1), 56. 10.1186/s13195-015-0139-9 26286145 PMC4543480

[B138] Wang C. Fan L. Khawaja R. R. Liu B. Zhan L. Kodama L. (2022). Microglial NF-κB drives tau spreading and toxicity in a mouse model of tauopathy. Nat. Commun. 13 (1), 1969. 10.1038/s41467-022-29552-6 35413950 PMC9005658

[B139] Wang P. L. Yim A. K. Y. Kim K. W. Avey D. Czepielewski R. S. Colonna M. (2020). Peripheral nerve resident macrophages share tissue-specific programming and features of activated microglia. Nat. Commun. 11 (1), 2552. 10.1038/s41467-020-16355-w 32439942 PMC7242366

[B140] Wang M. Liu M. Ma Z. (2023). Cannabinoid type 2 receptor activation inhibits MPP+-Induced M1 differentiation of microglia through activating PI3K/Akt/Nrf2 signal pathway. Mol. Biol. Rep. 50 (5), 4423–4433. 10.1007/s11033-023-08395-4 36977807

[B141] Wang P. Yang P. Qian K. Li Y. Xu S. Meng R. (2022). Precise gene delivery systems with detachable albumin shell remodeling dysfunctional microglia by TREM2 for treatment of alzheimer's disease. Biomaterials 281, 121360. 10.1016/j.biomaterials.2021.121360 34991033

[B142] Wang W. Li Y. Ma F. Sheng X. Chen K. Zhuo R. (2023). Microglial repopulation reverses cognitive and synaptic deficits in an alzheimer's disease model by restoring BDNF signaling. Brain Behav. Immun. 113, 275–288. 10.1016/j.bbi.2023.07.011 37482204

[B143] Whiting Z. M. Yin J. de la Harpe S. M. Vernall A. J. Grimsey N. L. (2022). Developing the cannabinoid receptor 2 (CB2) pharmacopoeia: past, present, and future. Trends Pharmacol. Sci. 43 (9), 754–771. 10.1016/j.tips.2022.06.010 35906103

[B144] Willis E. F. MacDonald K. P. A. Nguyen Q. H. Garrido A. L. Gillespie E. R. Harley S. B. R. (2020). Repopulating microglia promote brain repair in an IL-6-Dependent manner. Cell 180 (5), 833–846 e16. 10.1016/j.cell.2020.02.013 32142677

[B145] Wu J. Bie B. Yang H. Xu J. J. Brown D. L. Mohamed N. (2013). Activation of the CB2 receptor system reverses amyloid-induced memory deficiency. Neurobiol. Aging 34 (3), 791–804. 10.1016/j.neurobiolaging.2012.06.011 22795792

[B146] Xia Y. Zhang G. Kou L. Yin S. Han C. Hu J. (2021). Reactive microglia enhance the transmission of exosomal alpha-synuclein *via* toll-like receptor 2. Brain 144 (7), 2024–2037. 10.1093/brain/awab122 33792662

[B147] Xu K. Wu Y. Tian Z. Xu Y. Wu C. Wang Z. (2023). Microglial cannabinoid CB2 receptors in pain modulation. Int. J. Mol. Sci. 24 (3), 2348. 10.3390/ijms24032348 36768668 PMC9917135

[B148] Yang Z. Liu B. Yang L. E. Zhang C. (2019). Platycodigenin as potential drug candidate for alzheimer's disease *via* modulating microglial polarization and neurite regeneration. Molecules 24 (18), 3207. 10.3390/molecules24183207 31487775 PMC6767002

[B149] Yang Y. Mou B. Zhang Q. R. Zhao H. X. Zhang J. Y. Yun X. (2023). Microglia are involved in regulating histamine-dependent and non-dependent itch transmissions with distinguished signal pathways. Glia 71 (11), 2541–2558. 10.1002/glia.24438 37392090

[B150] Yoo Y. Neumayer G. Shibuya Y. Mader M. M. Wernig M. (2023). A cell therapy approach to restore microglial Trem2 function in a mouse model of alzheimer's disease. Cell Stem Cell 30 (8), 1043–1053 e6. 10.1016/j.stem.2023.07.006 37541210

[B151] Youssef D. A. El-Fayoumi H. M. Mona F. M. (2019). Beta-caryophyllene protects against diet-induced dyslipidemia and vascular inflammation in rats: involvement of CB2 and PPAR-γ receptors. Chemico-Biological Interact. 297, 16–24. 10.1016/j.cbi.2018.10.010 30343038

[B152] Yu H. Liu X. Chen B. Vickstrom C. R. Friedman V. Kelly T. J. (2021). The neuroprotective effects of the CB2 agonist GW842166x in the 6-OHDA mouse model of parkinson’s disease. Cells 10 (12), 3548. 10.3390/cells10123548 34944056 PMC8700250

[B117] Yu S.-J. Reiner D. Shen H. Wu K.-J. Liu Q.-R. Wang Y. (2015). Time-dependent protection of CB2 receptor agonist in stroke. PLOS ONE 10 (7), e0132487. 10.1371/journal.pone.0132487 26186541 PMC4505877

[B153] Zarruk J. G. Fernández-López D. García-Yébenes I. García-Gutiérrez M. S. Vivancos J. Nombela F. (2012). Cannabinoid type 2 receptor activation downregulates stroke-induced classic and alternative brain Macrophage/Microglial activation concomitant to neuroprotection. Stroke 43 (1), 211–219. 10.1161/STROKEAHA.111.631044 22020035

[B154] Zhang H. Y. Shen H. Jordan C. J. Liu Q. R. Gardner E. L. Bonci A. (2019). CB2 receptor antibody signal specificity: correlations with the use of partial CB2-knockout mice and anti-rat CB2 receptor antibodies. Acta Pharmacol. Sin. 40 (3), 398–409. 10.1038/s41401-018-0037-3 29967455 PMC6460367

[B155] Zhang C. Raveney B. Takahashi F. Yeh T. W. Hohjoh H. Yamamura T. (2023). Pathogenic microglia orchestrate neurotoxic properties of eomes-expressing helper T cells. Cells 12 (6), 868. 10.3390/cells12060868 36980209 PMC10047905

[B156] Zheng T. Zhang Z. (2021). Activated microglia facilitate the transmission of alpha-synuclein in Parkinson's disease. Neurochem. Int. 148, 105094. 10.1016/j.neuint.2021.105094 34097990

[B157] Zhou R. Chen S. H. Zhao Z. Tu D. Song S. Wang Y. (2023). Complement C3 enhances LPS-Elicited neuroinflammation and neurodegeneration *via* the Mac1/NOX2 pathway. Mol. Neurobiol. 60 (9), 5167–5183. 10.1007/s12035-023-03393-w 37268807 PMC10415527

[B158] Ziring D. Wei Bo Velazquez P. Schrage M. Buckley N. E. Braun J. (2006). Formation of B and T cell subsets require the cannabinoid receptor CB2. Immunogenetics 58 (9), 714–725. 10.1007/s00251-006-0138-x 16924491

[B159] Zrzavy T. Simon H. Wimmer I. Butovsky O. Weiner H. L. Lassmann H. (2017). Loss of ‘homeostatic’ microglia and patterns of their activation in active multiple sclerosis. Brain 140 (7), 1900–1913. 10.1093/brain/awx113 28541408 PMC6057548

