# Supplementary figures and images for: Novel floxed cannabinoid receptor 2 mouse line combines knockout capability with dual fluorescent reporters
Source: Front Pharmacol. 2025 Nov 19;16:1682979. doi: 10.3389/fphar.2025.1682979 (PMC12672438; doi:10.3389/fphar.2025.1682979)

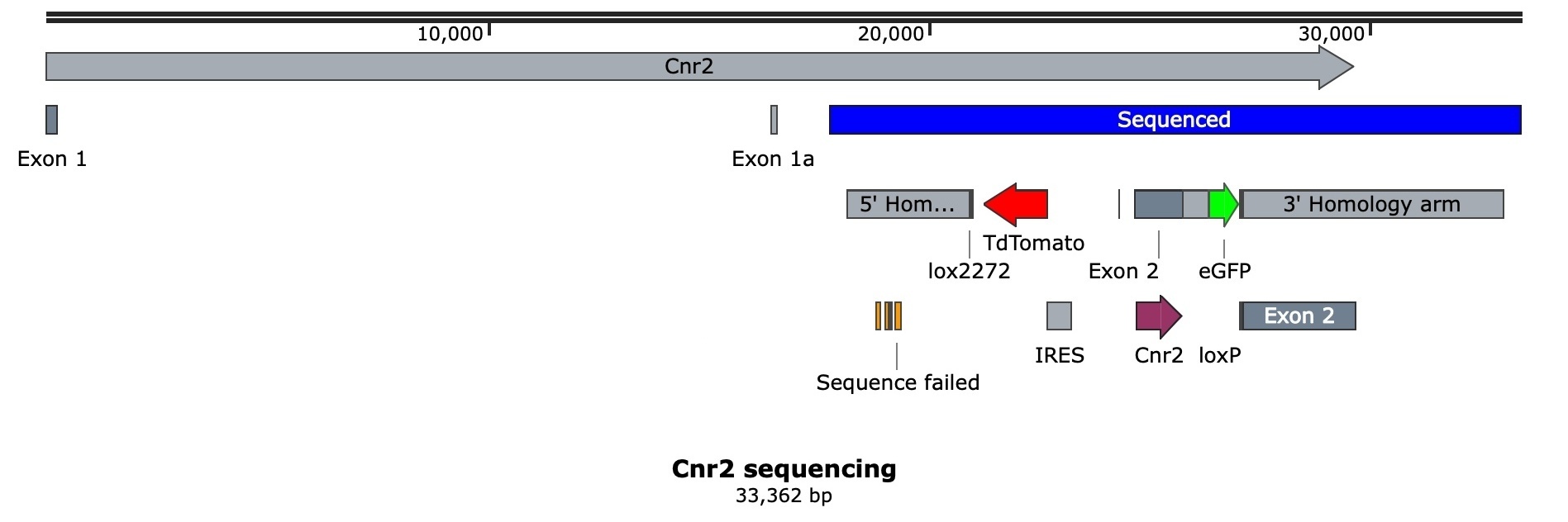

Supplement: Supplementary file 1 [file Image3.jpeg]

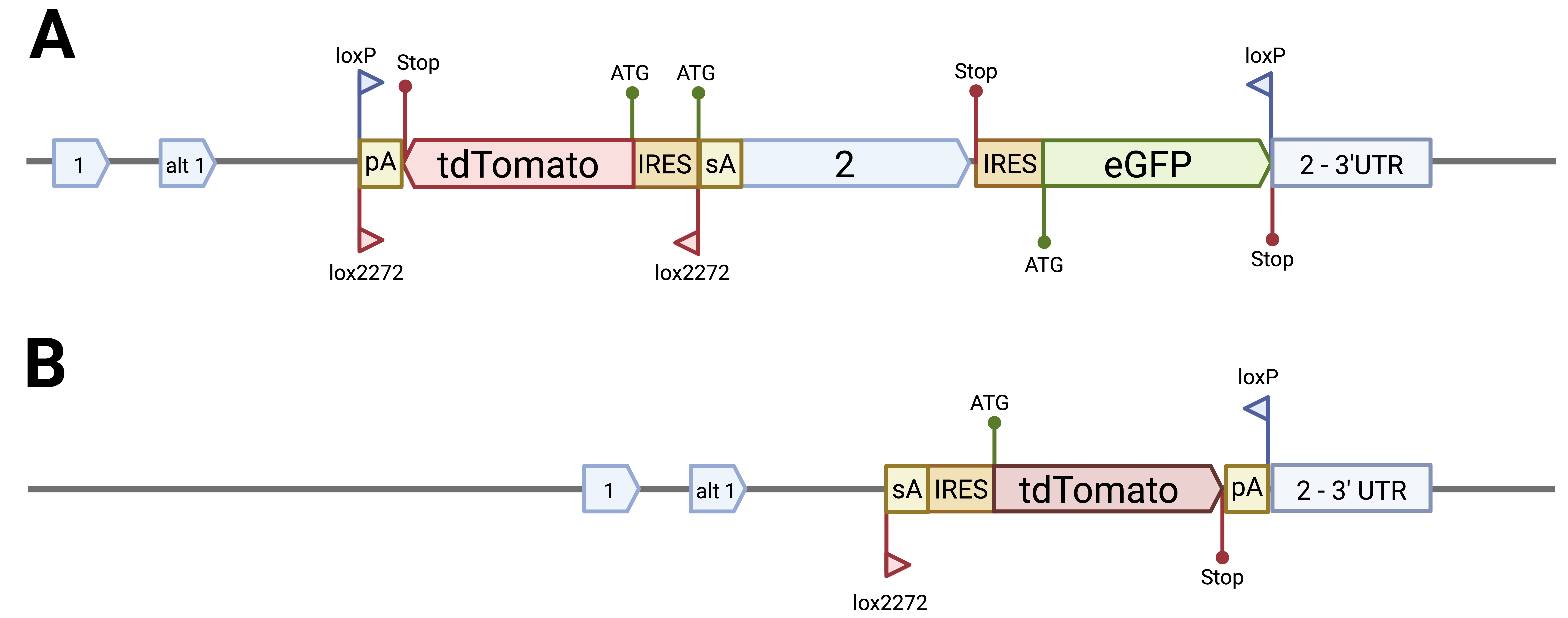

Supplement: Supplementary file 2 [file Image1.jpeg]

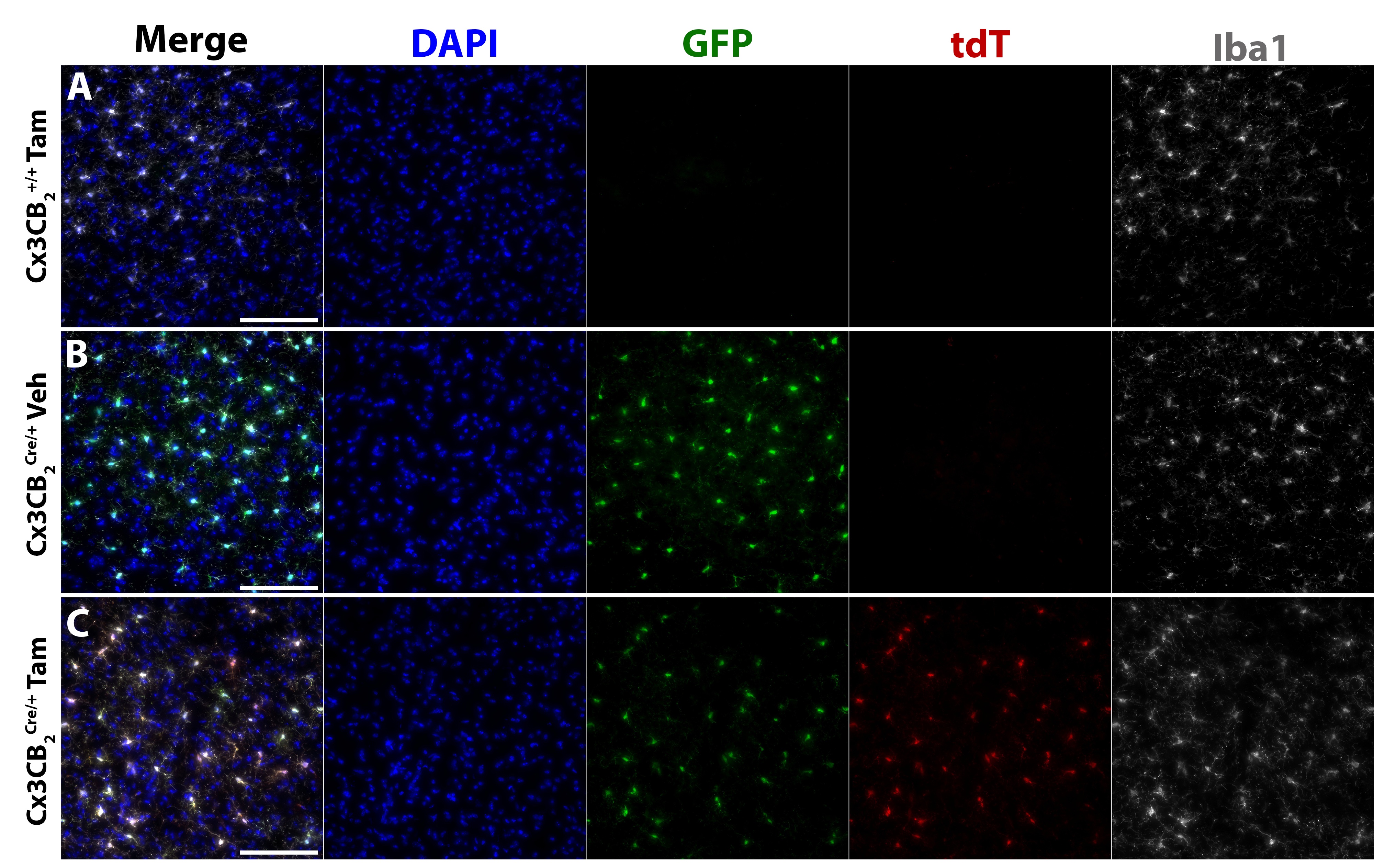

Supplement: Supplementary file 3 [file Image4.jpeg]

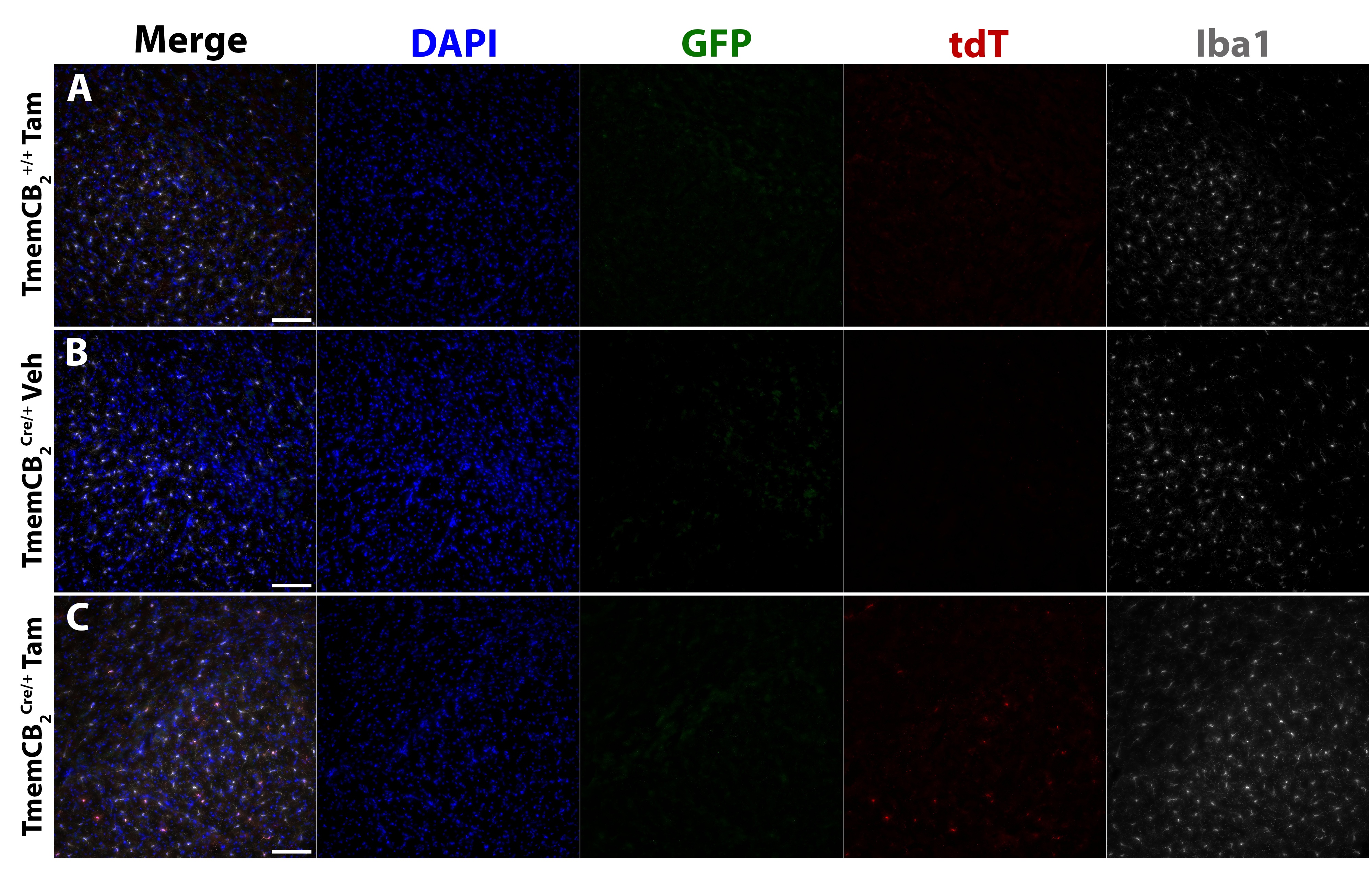

Supplement: Supplementary file 4 [file Image7.jpeg]

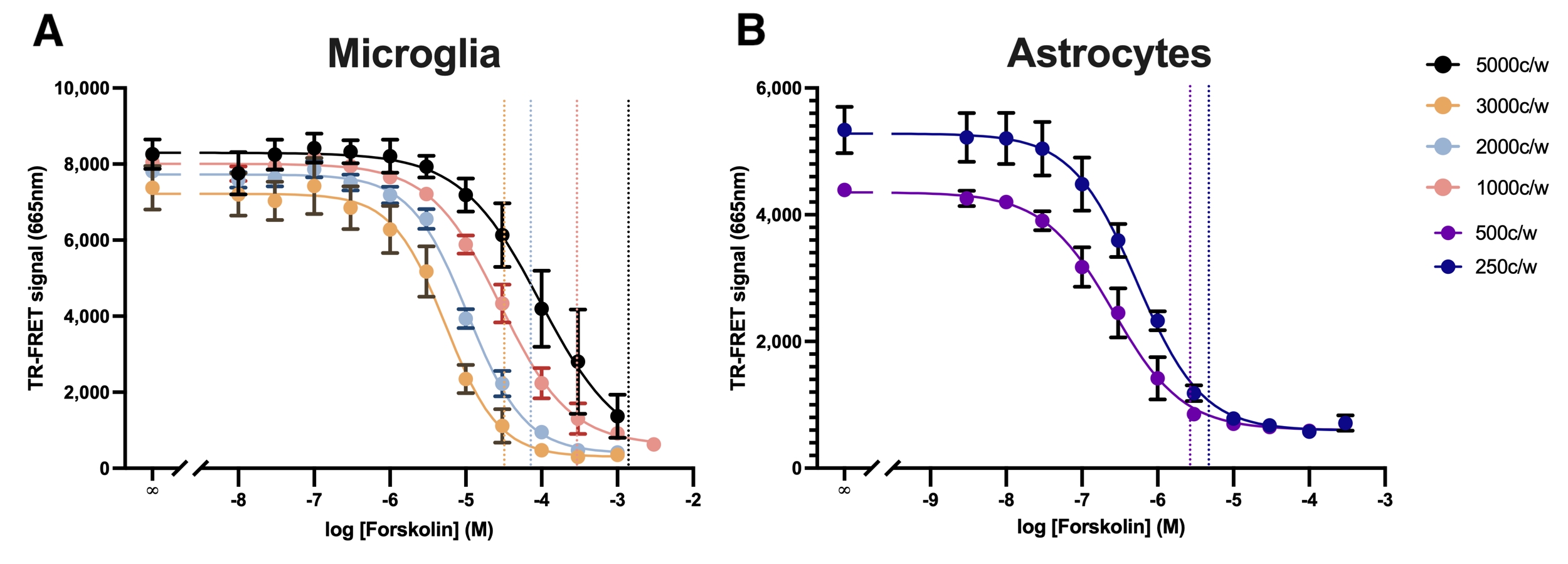

Supplement: Supplementary file 5 [file Image2.jpeg]

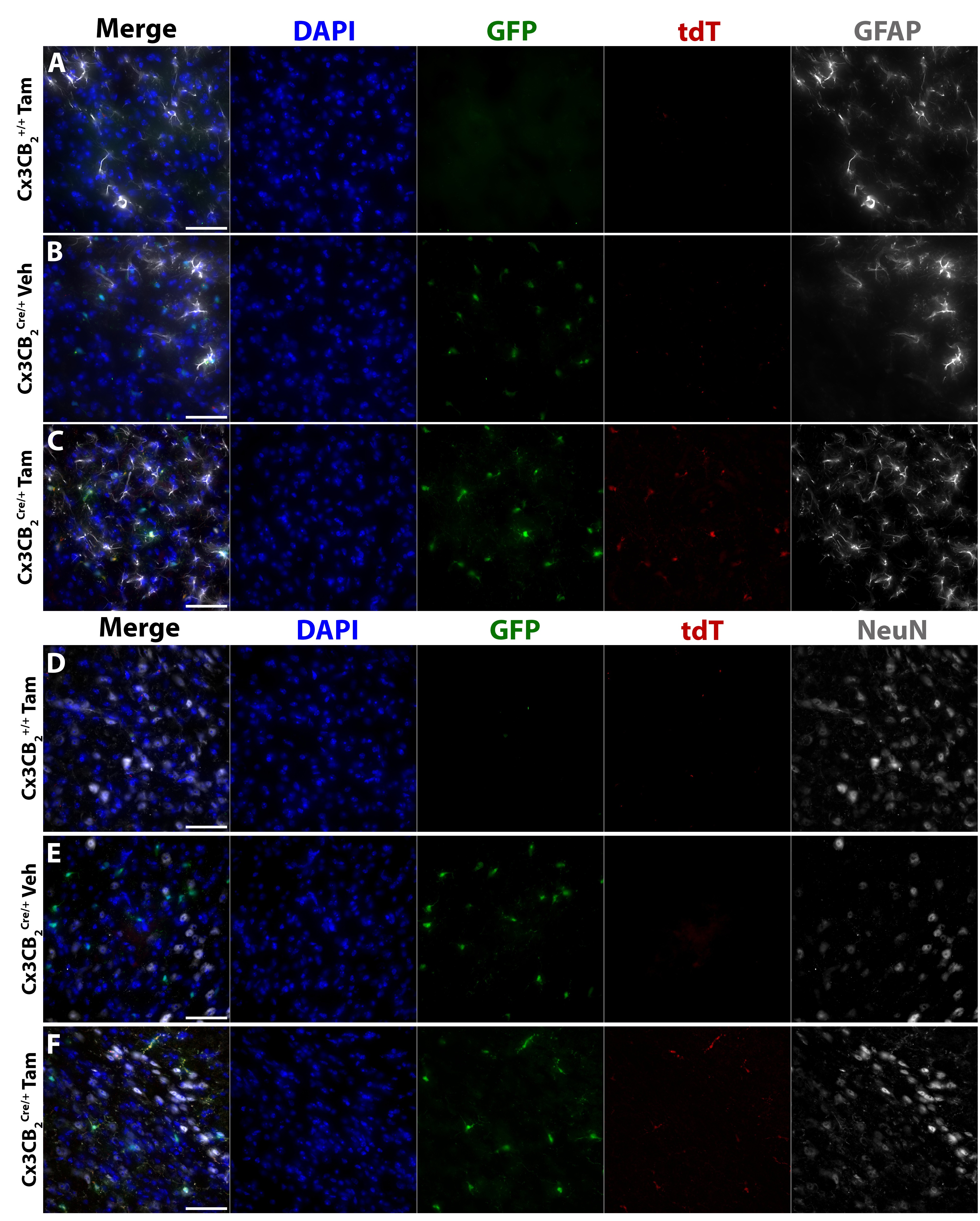

Supplement: Supplementary file 6 [file Image5.jpeg]

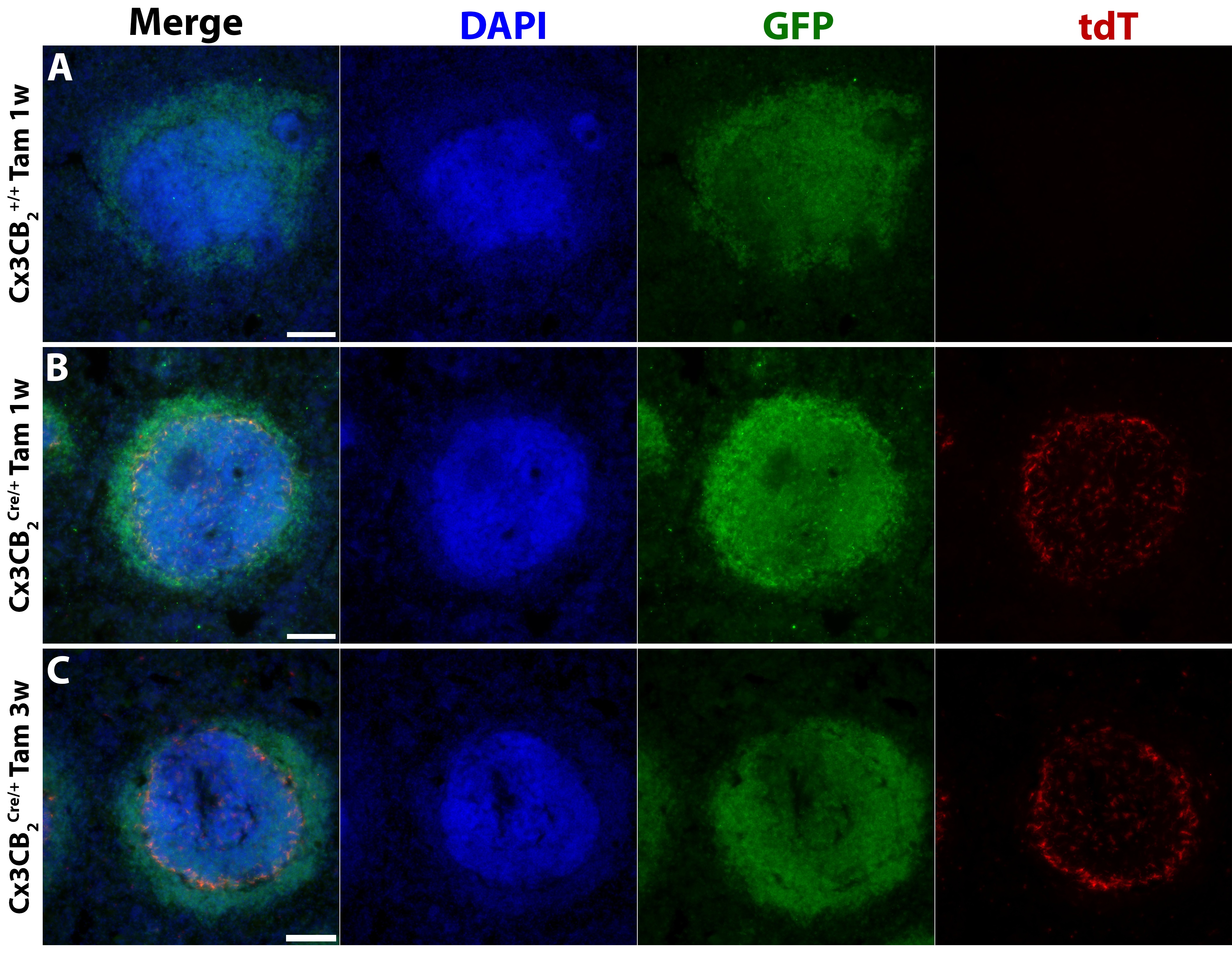

Supplement: Supplementary file 8 [file Image6.jpeg]
