# Supplementary material for: Novel floxed cannabinoid receptor 2 mouse line combines knockout capability with dual fluorescent reporters
Source: Front Pharmacol. 2025 Nov 19;16:1682979. doi: 10.3389/fphar.2025.1682979 (PMC12672438; doi:10.3389/fphar.2025.1682979)
Supplement: Supplementary file 7 [file DataSheet1.docx]

Supplementary Material

Supplementary Table 1: Primer sequences and PCR Conditions for genotyping

| Target | Source | Sequence 5’ → 3’ | Type |  | STEP | TIME (s) | TEMP ℃ |
| --- | --- | --- | --- | --- | --- | --- | --- |
| *Tmem119*^CreERT2^ | JAX 34019 | ATC GCA TTC CTT GCA AAA GT | Mutant Fwd |  | Denature | 30 | 98 |
|  |  | CAG TAT GTG GGG TCA CTG AAG A | Wild-type Fwd |  | 10 Cycles | 10 | 98 |
|  |  | ACT TGG GGA GAT GTT TCC TG | Common Rev |  |  | 30 | 65-55 touchdown |
| *Cx3cr1^C^*^reERT2^ | JAX 24463 | CGG TTA TTC AAC TTG CAC CA | Mutant Rev |  |  | 30 | 72 |
|  |  | AGG ATG TTG ACT TCC GAG TTG | Wild-type Rev |  | 25 Cycles | 10 | 98 |
|  |  | AAG ACT CAC GTG GAC CTG CT | Common Fwd |  |  | 30 | 55 |
| *Cnr2-*flx | Ozgene | GATCCCAATAAAAGCTAGCGGAAG | Mutant Fwd |  |  | 30 | 72 |
|  |  | TTTTGTGTGTGCTGGTGTAGTG | Wild-type Fwd |  | Final | 120 | 72 |
|  |  | TTTGGCTTCTTCTACTGGAGCTG | Common Rev |  | Hold | ∞ | 4 |

Supplementary Table 2: Antibody resource table

| *NAME* | HOST | CONC | SOURCE | Cat# |
| --- | --- | --- | --- | --- |
| Anti-Iba1 | Rabbit | 1:1000 | Wako | 019-19741 |
| Anti-Chicken IgY Secondary, AlexaFluor 488 | Donkey | 1:250 | Thermo Fisher | A78948 |
| Anti-Glial Fibrillary Acidic Protein | Rabbit | 1:300 | Agilent | Z033401 |
| Anti-Goat IgG Secondary Antibody, Alexa Fluor Plus 647 | Donkey | 1:250 | Thermo Fisher | A32849 |
| Anti-Green Fluorescent Protein | Chicken | 1:1000 | Aves Labs | GFP-1020 |
| Anti-NeuN, clone A60 | Mouse | 1:500 | Sigma-Aldrich | MAB377 |
| Anti-Rabbit IgG Secondary, AlexaFluor 555 | Donkey | 1:250 | Thermo Fisher | A32794 |
| Anti-RFP (pre-adsorbed) | Rabbit | 1:1000 | Rockland | 600-401-379 |

| Supplementary Table 3: Key resources table | | | | |
| --- | --- | --- | --- | --- |
| RESOURCE/PRODUCT | | SOURCE | | IDENTIFIER |
| Mouse Models | | | | |
| C57BL/6JAusb | | Australian BioResources | | RRID:MGI:6200612 |
| CB_2_flx | | Ozgene Pty Ltd | | N/A |
| Cx3cr1^Cre/ERT2^ | | The Jackson Laboratory | | RRID:IMSR_JAX:021160 |
| Tmem119^Cre/ERT2^ | | The Jackson Laboratory | | RRID:IMSR_JAX:031820 |
| Cx3cr1-CB_2_ | | Novel | | N/A |
| Tmem119-CB_2_ | | Novel | | N/A |
| Chemicals & Reagents | | | | |
| 100 bp DNA Ladder | | Thermo Fisher | | 15628019 |
| BlueJuice Gel Loading Buffer (10X) | | Thermo Fisher | | 10816015 |
| Bovine Serum Albumin Bovostar | | Bovogen | | BSAS-NZ |
| Bupivacaine HCL 0.5% | | Pfizer | | BUPI I 3 |
| Cultrex | | BioTechne | | 3432-005-01 |
| D-(+)-Glucose | | Sigma-Aldrich | | G8270 |
| DAPI | | Thermo Fisher | | D1306 |
| Dimethyl sulfoxide | | Sigma-Aldrich | | D8418 |
| DMEM/F-12 | | Thermo Fisher | | 11320033 |
| DPBS | | Thermo Fisher | | 14190250 |
| dTNPs | | New England Biolabs | | N0447L |
| Forskolin | | Sigma-Aldrich | | 344270 |
| Glycerol Gelatin | | Sigma-Aldrich | | GG1 |
| HBSS | | Thermo Fisher | | 14175103 |
| Hu308 | | Tocris | | 3088 |
| Ketamine | | Mavlab Animal Health | | Ketamev 100 |
| Lance Ultra cAMP kit | | PerkinElmer | | TRF0263 |
| Lipopolysaccharide | | Sigma-Aldrich | | L3024 |
| O.C.T. Embedding Medium | | Scigen | | 4586 |
| Paraformaldehyde, prilled | | Sigma-Aldrich | | 441244 |
| Penicillin-Streptomycin | | Sigma-Aldrich | | 441244 |
| Poly-D-lysine hydrobromide | | Sigma-Aldrich | | P7405 |
| Q5 High-Fidelity DNA Polymerase | | New England Biolabs | | M0491 |
| Q5 Reaction Buffer Pack | | New England Biolabs | | B9027S |
| RIPA Buffer | | Sigma-Aldrich | | R0278 |
| RT-PCR Grade Water | | Thermo Fisher | | AM9935 |
| Sodium Azide | | Thermo Fisher | | AJA1222-500 |
| Sucrose | | Sigma-Aldrich | | S9378 |
| SYBR Safe DNA Gel Stain | | Thermo Fisher | | S33102 |
| Tamoxifen | | Sigma-Aldrich | | T5648 |
| Triton X-100 | | Sigma-Aldrich | | T8787 |
| Trypsin-EDTA (0.5%) | | Thermo Fisher | | 15400054 |
| Tween 80 | | Sigma-Aldrich | | P6224 |
| UltraPure Agarose | | Thermo Fisher | | 16500100 |
| Xylazine hydrochloride | | Troy Lab | | ilium Xylazil 20 |
| Equipment/other | | | | |
| 2.0µL, Neuros Syringe | | Hamilton | | 65459-01 |
| Brightfield microscope | | Zeiss | | Axio Imager A1 |
| Countess II FL | | Thermo Fisher | | AMQAF2000 |
| Cryostat | | Leica | | CM3050 S |
| Fluorescent Microscope | | Zeiss | | Axio Imager Z2 |
| Microplate Reader | | BMG LabTech | | PHERAstar FSX |
| Nunc Lab-Tek Chamber Slide System | | Thermo Fisher | | 154941PK |
| Openfield | | Med Associates | | MED-OFAS-MS |
| Rotarod | | Ugo Basile | | 47650 |
| Thunder Imaging System | | Leica | |  |
| Wide Mini-Sub Cell GT Cell | | Bio-Rad | | 1704405 |
| Software | | | | |
| Activity Monitor | Med Associates Inc. | | SOF-812 | |
| Fiji | Schindelin, Arganda-Carreras (157) | | www.fiji.sc/ | |
| Prism 9 | Graphpad | | www.graphpad.com/scientific-software/prism/ | |
| Stereo Investigator | MBF Bioscience | | www.mbfbioscience.com/products/stereo-investigator | |
